# PPARα Ameliorates Doxorubicin-Induced Cardiotoxicity by Reducing Mitochondria-Dependent Apoptosis *via* Regulating MEOX1

**DOI:** 10.3389/fphar.2020.528267

**Published:** 2020-10-08

**Authors:** Wei Wang, Qin Fang, Zhihao Zhang, Daowen Wang, Lujin Wu, Yan Wang

**Affiliations:** ^1^Division of Cardiology, Department of Internal Medicine, Tongji Hospital, Tongji Medical College, Huazhong University of Science and Technology, Wuhan, China; ^2^Hubei Key Laboratory of Genetics and Molecular Mechanisms of Cardiological Disorders, Huazhong University of Science and Technology, Wuhan, China

**Keywords:** doxorubicin, PPARα, mitochondria, apoptosis, cardiotoxicity

## Abstract

Doxorubicin (DOX), a chemotherapeutic drug widely used in the clinical setting, is known to cause serious cardiotoxicity and greatly reduces the survival rate as well as quality of life of patients receiving chemotherapy. Peroxisome proliferation activated receptor α (PPARα) is a type of ligand activated receptor of the nuclear hormone receptor family that regulates multiple gene expression. Several studies have shown that PPARα has anti-apoptotic and cardio-protective effects. However, its role in DOX-induced cardiotoxicity is rarely reported. In this study, we observed decreased expression of PPARα in the heart of tumor-bearing mice already treated with DOX; however, no such phenomenon was observed in tumor tissues. Next, we observed that the PPARα agonist, fenofibrate (FENO), had no effect on tumor progression; however, it enhanced cardiac function in tumor-bearing mice treated with DOX. Subsequently, recombinant adeno-associated virus serotype 9 (rAAV9) was used to manipulate the expression of PPARα in the heart of DOX-induced mice. Our results showed that PPARα gene delivery reduced cardiac dysfunction and mitochondria-dependent apoptosis in DOX-induced mice. Furthermore, we found that PPARα directly regulated the expression of mesenchyme homeobox 1 (MEOX1). Most importantly, the cardioprotective effects of PPARα could be neutralized by knocking down MEOX1. In summary, PPARα plays a vital role in DOX-induced cardiotoxicity and is a promising treatment target.

## Introduction

Doxorubicin (DOX), originally obtained from *Streptomyces* mutants, is a widely used chemotherapeutic drug. It has a high affinity for DNA and forms DOX-DNA complexes, resulting in mitotic disorder and DNA double strand breakage (Renu et al., 2018). DOX is mainly used in chemotherapy for solid tumors and hematological malignancies, and has been reported to significantly improve the survival rate of patients with cancer. However, the clinical application of DOX is limited by its concentration-dependent, irreversible, and progressive cardiotoxicity (Songbo et al., 2019). DOX is known to cause cardiotoxicity in adults and children at cumulative doses greater than 400 to 700 mg/m^2^, and 300 mg/m^2^, respectively (Renu et al., 2018; Songbo et al., 2019). The incidence of cardiotoxicity is 5% when the dose reaches 400 mg/m^2^ in adults and 26% when it reaches 550 mg/m^2^, while the incidence of cardiotoxicity is as high as 48% when it reaches 700 mg/m^2^ (Renu et al., 2018). One of the most serious complications of DOX is cardiomyopathy, which occurs in patients 4 to 20 years after DOX treatment, with varying degrees of concentration dependence (Singal and Iliskovic, 1998). This property is called as “dose memory.” The important mechanisms of cardiotoxicity induced by DOX include the production of reactive oxygen species (ROS), weakness of antioxidant system, and dysfunction of mitochondria; resulting in chronic myocardial fiber loss and vacuolation, and eventually leading to apoptosis, necrosis, autophagy, and senescence (Lebrecht et al., 2003; Zhang et al., 2009; Songbo et al., 2019). It is worth mentioning that mitochondrial dysfunction induced by ROS initiates the endogenous apoptotic pathway, which suggests that the damage of structure and function of mitochondria may play a key role in DOX-induced cardiotoxicity. Despite the continuous synthesis of new anthracycline drugs, DOX still has advantages with respect to its anti-tumor effects, and is thus irreplaceable at present (Zhang et al., 2009; Sinha et al., 2013; Dominic et al., 2014). However, the mechanism of DOX-induced cardiotoxicity is very complex and requires further investigation.

Peroxisome proliferation activated receptor α (PPARα), a kind of nuclear hormone receptor and regarded as a transcription factor, is highly expressed in the heart (Robinson and Grieve, 2009; Pawlak et al., 2015). Fatty acids, leukotriene derivatives, and very low-density lipoprotein hydrolysates are endogenous high affinity ligands, while exogenous ligands include fibrates, such as fenofibrate (FENO) (Packard, 1998; Vosper et al., 2002; Fidaleo et al., 2014). The ligand binds to PPARα in the cytoplasm and then translocates into the nucleus, followed by forming a heterodimer with retinoic acid receptor (RXR), binding to the PPAR response element and finally regulating the expression of multiple target genes (Robinson and Grieve, 2009; Wilding, 2012; Grygiel-Gorniak, 2014; Pawlak et al., 2015). Generally, PPARα is thought to be involved in lipid metabolism. However, over delivery of fatty acids to the cardiac tissues increases cardiac exposure to lipid toxicity, leading to decreased expression of PPARα, oxidative stress, and mitochondrial remodeling (Elezaby et al., 2015). Lipid peroxidation results in the weakening of the anti-oxidation system in the cells, which leads to mitochondrial dysfunction and further increases organ damage (Begriche et al., 2006). In addition, knock-out of the PPARα of mice or the inhibition of PPARα increases sensitivity to oxidative stress (Abdelmegeed et al., 2009). During myocardial ischemia-reperfusion and pressure-induced myocardial hypertrophy, PPARα is down-regulated. However, this is considered harmful because this process is accompanied with the production of ROS and decrease in ATP production. In addition, there is a causal relationship between the production of ROS and the downregulation of PPARα in myocardial hypertrophy caused by hypertension, and this ultimately leads to myocardial energy impairment. Therefore, the activation of PPARα has been shown to be beneficial in these pathological conditions (Vosper et al., 2002; Robinson and Grieve, 2009). Of note, DOX-DNA complexes that can inhibit the expression of PPARα are formed in the nucleus. In addition, DOX enters the mitochondria and then binds to the topoisomerase 2β (TOP2β). These two conditions increase the production of ROS and eventually lead to apoptosis (Zhang et al., 2012; Brown et al., 2015). Therefore, there is some mutual regulatory relationship among PPARα, oxidative stress and mitochondrial function.

However, the role of PPARα in DOX-induced cardiotoxicity has not yet been fully clarified. In the present study, we demonstrated that the cardioprotective effect of PPARα was achieved by reducing oxidative stress and mitochondria-dependent apoptosis.

## Materials and Methods

### Cell Culture and Treatment

H9C2 cells, HEK293T cells, A549 cells, MDA-MB-231 cells, and TC-1 cells were obtained from the ATCC, and were cultured in 10% FBS Dulbecco’s modified Eagle’s medium (DMEM) at 37°C, with 5% CO_2_. When cells reached about 80% confluence, they were treated with a corresponding concentration of DOX. After 24 h, the cells were collected and used for follow-up detection.

### Mice

The animal experiments were performed according to the Guide for the Care and Use of Laboratory Animals, published by the United States National Institutes of Health (NIH Publication No. 85-23, revised 1996). All experimental procedures were approved by the Experimental Animal Research Committee of Tongji Medical College, Huazhong University of Science and Technology, Wuhan, China.

### Animal Experiment 1

To observe the effect of PPARα activation on tumor growth *in vivo*, we used the mouse tumor cell line, TC-1 cells, to carry out subcutaneous implanting in 6- to 8-week-old male C57BL/6 mice. TC-1 cells (1 ×  10^6^) were injected subcutaneously into each mouse. Tumor size was uniform among the groups at day 1. Subsequently, the mice were randomly divided into four groups: Control, FENO (100 mg/kg/d, p.o), DOX (24 mg/kg, i.p), and DOX+FENO. Tumor size was measured once every two days with a digital calliper. The shape of tumors was regarded as an elliptical sphere, and their volume were calculated according to the following formula: V = (width)^2^ × length × π/6. When the tumor was 2 cm in any dimension, the mice would be euthanized.

### Animal Experiment 2

Male C57BL/6 mice (6–8 weeks old) were randomly divided into six groups: Control, rAAV9-GFP, rAAV9-PPARα, DOX (24 mg/kg, i.p), DOX+rAAV9-GFP and DOX+rAAV9-PPARα. Then, rAAV9 (1 × 10^11^ vector genomes per mouse) was injected into the tail veins and DOX treatment (**Figure 3A**) was initiated 2 weeks later.

### Western Blotting Analysis

Total protein was extracted using RIPA lysis buffer, supplemented with protease inhibitor. Proteins were subjected to SDS-PAGE gels, and were then transferred to PVDF membranes. Next, the membranes were blocked in evaporated milk at room temperature for 1 h. Primary antibodies were incubated with membranes overnight at 4°C. After washing with Tris-buffered saline with tween, the membranes were incubated with horseradish peroxidase conjugated secondary antibodies. Finally, electrochemiluminescence system was used to detect the bands. Primary antibodies for immunoblotting are listed in **Supplementary Table 1**.

### Total RNA Isolation, Reverse Transcriptional Polymerase Chain Reaction and Real-Time Polymerase Chain Reaction (RT-PCR)

Total RNA was isolated with RNAiso Plus (Cat# 9109, Takara Biomedical Technology, Beijing, China), according to the manufacturer’s instructions and reverse-transcribed by PrimeScript RT reagent Kit with gDNA Eraser (Cat# RR047A, Takara Biomedical Technology). RT-PCR was performed to quantify mRNA levels using TB Green Premix Ex Taq II (Tli RNaseH Plus, Cat# RR820A, Takara Biomedical Technology). The primers used in this study are listed in **Supplementary Table 2**.

### Annexin V-FITC/PI Apoptosis Detection

For the detection of apoptosis, Annexin V-FITC/PI apoptosis detection kit (Cat# 556547, BD Pharmingen, NJ, USA) was used. All operations referred to the instructions of the manufacturer. Finally, quantitative analysis was performed by a FACStar Plus flow cytometer (BD, NJ, USA).

### Cell Viability Assay

Cell counting kit-8 (CCK8, Cat# AR1160, Boster Biological Technology, Wuhan, China) was used to detect cell viability, in accordance with the protocol provided by the manufacturer. Briefly, logarithmic phase cells were collected followed by adjusting the cell number. Then, approximately 10,000 cells were added to each well of the plate. After culturing at 37°C, with 5% CO_2_, for 24 h, the cells were treated using DOX, supplemented with or without Wy-14643. After incubating at 37°C in the dark for 1 h, absorbance was detected by enzyme-labeled instrument at 450 nm.

### Package of Recombinant Adeno-Associated Virus Serotype 9 (rAAV9)

RAAV9 system, which was a present from Dr. Xiao (University of North Carolina at Chapel Hill), was used as the vector to manipulate the expression of PPARα in the heart. We co-transfected the recombinant plasmid containing mouse PPARα or enhanced green fluorescent protein (EGFP) with the packaging plasmid into HEK293T cells. The preparation and purification of the virus has been described previously (Dai et al., 2017).

### Echocardiography and Hemodynamic Monitoring

After isoflurane (1–2%) anesthesia in mice, high-resolution probe and ultrasound imaging system (Visual SonicsVevo-1100, Visual Sonics Inc., Toronto, Canada) were used to detect the long-axis section and the short-axis section of the heart, respectively. Left ventricular hemodynamics was performed by Millar Catheter System, as described previously (Wu et al., 2018).

### Hematoxylin and Eosin (H&E) Staining and Masson’s Trichrome Staining

The heart was fixed with 4% paraformaldehyde, embedded in paraffin, and cut into 5 µm sections. Subsequently, H&E staining and Masson′s trichrome staining were performed to assess the myocardial cross-sectional area and fibrosis area using Image Pro Plus (Version 6.0, Media Cybernetics, Inc., Rockville, MD).

### Dihydroethidium (DHE) Fluorescent Staining and Immunofluorescence Staining

DHE was used to detect ROS content in fresh frozen LV sections (6 μm thick), as described previously (Hu et al., 2019). The sections were stained with DHE (5 μM, Cat# S0063, Beyotime Biotechnology, Nanjing, China) for 30 min at 37°C in the dark. Red fluorescence was detected by a fluorescence microscope (Olympus IX53, Tokyo, Japan). For immunofluorescence staining, cardiac sections were incubated with 1:100 dilution of PPARα (Cat# bsm-51405M, Bioss Antibodies, Beijing, China) and MEOX1 (Cat# ab105349, Abcam) antibody. Finally, they were stained with Cy3-conjugated (goat anti-mouse, Cat# BA1031, Boster Biological Technology) and Alexa Fluor555-conjugated (donkey anti-rabbit, Cat# ab150074, Abcam) secondary antibody for fluorescent imaging.

### Mitochondrial DNA (mtDNA) Copy Number Quantification

Total genomic DNA was extracted by TIANamp Genomic DNA Kit (Cat# DP304, Tiangen Biotech, Beijing, China). The relative expression of mtDNA was detected by RT-PCR using TB Green Premix Ex Taq II, as described in previous studies (Sun et al., 2013; Malik et al., 2016).

### Detection of ATP Content

ATP content was measured by the ATP bioluminescent assay kit (Cat# S0026, Beyotime Biotechnology), according to the manufacturer′s instruction. The chemiluminescence was detected by a luminometer (TurnerBioSystems, CA, USA).

### Terminal Deoxynucleotidyl Transferase-Mediated dUTP-Biotin Nick End-Labeling (TUNEL) Assay

All procedures were carried out in accordance with the instructions of In Situ Cell Death Detection Kit (Cat# 11684795910, Roche Diagnostics, Laval, Canada). 4′,6-diamidino-2-phenylindole (DAPI) was used to recognize the nuclei. After staining, a fluorescence microscope was used to observe the ratio of TUNEL positive cells.

### Detection of Cellular ROS in Cardiomyocytes

Detection of ROS in H9C2 cells was performed using 2′,7′-dichlorofluorescein diacetate (DCFH-DA, Cat# S0033, Beyotime Biotechnology). All procedures were carried out in accordance with the manufacturer’s standard instructions. Briefly, the cells were incubated with DCFH-DA, which was diluted in a 1: 1000 ratio with serum-free medium, in the dark at 37°C for 40 min. Finally, the cells were washed three times with serum-free cell culture medium, followed by detection by flow cytometry.

### Mitochondrial Transmembrane Potential (ΔΨm) Assay

To determine mitochondrial transmembrane potential in H9C2 cells, 5,5′,6,6′-Tetrachloro-1,1′,3,3′-tetraethylbenzimidazolylcarbocyanine iodide (JC-1, Cat# C2006, Beyotime Biotechnology) was used. Detailed procedure can be found in the protocol of the product. In brief, H9C2 cells were incubated with JC-1 staining buffer for 30 min in the dark at 37°C. Then, the cells were rinsed twice with JC-1 rinsing buffer at 4°C. Finally, the cells were resuspended with phosphate-buffered saline. The fluorescence intensity was measured by flow cytometry.

### Microarray Data, Identification of Differentially Expressed Genes and PPARα Target Genes Prediction

The following data sets were obtained from Gene Expression Omnibus: GSE81448 (https://www.ncbi.nlm.nih.gov/geo/query/acc.cgi?acc=GSE81448), GSE59672 (https://www.ncbi.nlm.nih.gov/geo/query/acc.cgi?acc=GSE59672), and GSE23598 (https://www.ncbi.nlm.nih.gov/geo/query/acc.cgi?acc=GSE23598). These data sets contained global gene expression profiles in the hearts of control and DOX-induced mice. Intersectional analyses were performed using R project (v3.4.1). The finding criterion of differentially expressed genes was according to p value < 0.05. Genes in the DOX treatment group that changed more than twice as much as those in the control group were selected. Subsequently, the genes with different expression were screened by intersectional analysis. Bioinformatics websites PROMO (http://alggen.lsi.upc.es/cgi-bin/promo_v3/promo/promoinit.cgi?dirDB=TF_8.3) and LASAGNA (http://biogrid-lasagna.engr.uconn.edu/lasagna_search/) were used for predicting differentially expressed genes that may be regulated by PPARα (Messeguer et al., 2002; Farré et al., 2003; Lee and Huang, 2014).

### Dual Luciferase Assay

To determine whether PPARα regulates MEOX1 gene with its transcription activity, we constructed MEOX1 reporter plasmid using PGL3-promoterless vector. Human or rat MEOX1 promoter (upstream −2,000–0 bp of CDS) was inserted into the plasmid. In addition, homologous PPARα cDNA was inserted into PCDNA3.1 expression vector. These were co-transfected into HEK293T and H9C2 cells, respectively, to carry out luciferase reporter assay 48 h after transfection, using Renilla luciferase activity as the reference. Each reporter was repeated in at least three independent experiments.

### ChIP Assay

For ChIP assay, ChIP Assay Kit (Cat# P2078, Beyotime Biotechnology) and PCR purification Kit (Cat# D0033, Beyotime Biotechnology) were used. Primer sequences were as follows: #1 (−1734 to −1724 bp) F: 5′ CCTGGGCGACAGAACGAGAC 3′, R: 5′ GGGGTTTGAGGTTAGACGGGT 3′, and #2 (−1091 to −1081 bp) F: 5′ CTTTGGAAGCAGCCAGCAG 3′, R: 5′ GCAATTTCTTGGAGGACGACA 3′. Anti- PPARα was from Abcam (Cat# ab24509, UK), and IGg was from Cell Signaling Technology (Cat# 2729, MA, USA). All the procedures were the same, as described previously (Pan et al., 2016).

### Small-Interfering RNA (siRNA) Transfection

H9C2 cells were transfected with Si-MEOX1 (100 nM, similarly hereinafter) or Si-NC using Lipofectamine 2000 Transfection Reagent (Cat# 11668027, Invitrogen, CA, USA), according to the manufacturer′s instruction. Forty-eight hours later, the cells were pre-treated with or without Wy-14643 for 1 h, followed by DOX treatment for an additional 24 h. The cells were then collected for follow-up detection.

### Statistical Analysis

All data are represented as means ± SEM. Unpaired two-tailed Student’s t test for independent data of normal distribution and one-way ANOVA with Tukey’s test for multiple comparisons were performed in GraphPad Prism 5.0 (GraphPad Soft-ware, San Diego, CA, USA). Paired two-tailed t test was applied to ChIP assay. Statistical P values < 0.05 were considered significant.

## Results

### PPARα Expression Was Downregulated and Cell Survival Was Impaired in DOX-Treated Cardiomyocytes

**Figure 1:**
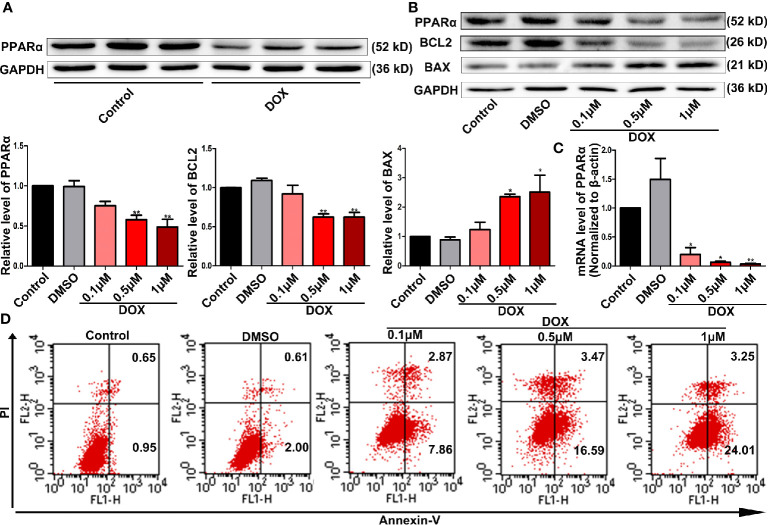
DOX decreased PPARα expression and impaired cell survival. **(A)** Representative image of Western blot for PPARα expression in the hearts from tumor-bearing mice treated with or without DOX, N = 3. **(B)** Expression levels of PPARα, BCL2, and BAX in H9C2 cells treated with different concentrations of DOX were quantified by Western blot. N = 4 independent experiments. **(C)** Messenger RNA level of PPARα in H9C2 cells treated with different concentrations of DOX. N = 4. **(D)** Representative images of flow cytometry results for apoptosis in H9C2 cells treated with different concentrations of DOX. N = 3. *P < 0.05 vs. Control; **P < 0.01 vs. Control.

In this study, we detected the expression of PPARα in the heart and tumor tissues of tumor-bearing mice treated with DOX. We found that PPARα expression decreased in heart tissues (**Figure 1A**) but did not change in the tumor tissues (**Figure S1A**). Therefore, we focused on the effects of PPARα on DOX-treated cardiomyocytes. Interestingly, DOX reduced the expression of PPARα in H9C2 cells, both at the protein and mRNA levels (**Figures 1B, C**). Next, we detected the cardiotoxicity of DOX under different doses and time points, by the CCK8 assay. We found that 0.5 and 1 μM of DOX significantly reduced cell viability at 24 h (**Figure S1B**). Similarly, cardiomyocyte apoptosis was significantly increased by DOX at concentrations of 0.5 and 1 μM (**Figure 1D** and **Figure S1C**). In addition, DOX increased the expression of the apoptosis protein BAX, whereas it decreased the expression of the anti-apoptosis protein BCL2 (**Figure 1B**). These data suggest that PPARα might play an important role in DOX-induced cardiotoxicity.

### PPARα Improved Cardiac Function in Tumor-Bearing Mice Treated With DOX Without Facilitating Tumor Progression

**Figure 2:**
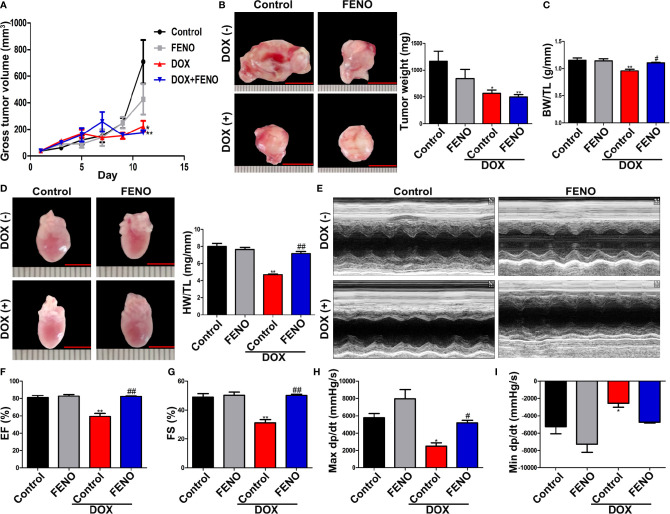
The activation of PPARα ameliorated cardiac function of tumor-bearing mice treated with DOX without promoting tumor growth. **(A)** Gross tumor volume of tumor-bearing mice treated with DOX with or without FENO at different time points. N = 6–7. **(B)** Representative images and weight of subcutaneous tumors in tumor-bearing mice. N = 6–7, bar = 10 mm. **(C)** Body weight/tibia length ratio of tumor-bearing mice in each group. N = 6–7. **(D)** Heart weight/tibia length ratio and corresponding representative pictures of tumor-bearing mice in each group. N = 6–7, bar = 5 mm. **(E)**. Representative echocardiography in M mode. **(F**, **G)** LVEF **(F)** and LVFS **(G)** of tumor-bearing mice treated with DOX with or without FENO. N = 6–7. **(H**, **I)** Max dp/dt **(H)** and min dp/dt **(I)** of tumor-bearing mice treated with DOX with or without FENO. N = 4–5. *P<0.05 vs. Control; **P<0.01 vs. Control; ^#^P<0.05 vs. DOX; ^##^P<0.01 vs. DOX.

Next, we tested the effects of PPARα activation combined with DOX therapy on tumor cells *in vitro* and *in vivo*. First, we observed that DOX significantly reduced the viability of tumor cells *in vitro*, although the application of the PPARα agonist alone did not affect the viability of tumor cells (**Figures S2A–C**). In vivo, DOX significantly inhibited tumor growth, alone and in combination with FENO. However, FENO alone did not show any obvious effects on tumor size (**Figure 2A**). Tumor weight was also significantly reduced by DOX unlike FENO (**Figure 2B**). Additionally, reductions in the body weight/tibia length ratio (BW/BL, **Figure 2C**) and heart weight/tibia length ratio (HW/HL, **Figure 2D**) were observed due to DOX-induced cardiotoxicity. Systolic and diastolic functions of the heart were also significantly damaged (**Figures 2E–I**). Interestingly, the application of FENO significantly ameliorated these injuries. Taken together, these results show that activation of PPARα did not affect the growth of tumor but relieved DOX-induced cardiotoxicity.

### Overexpression of PPARα Improved Cardiac Function in DOX-Induced Mice

**Figure 3:**
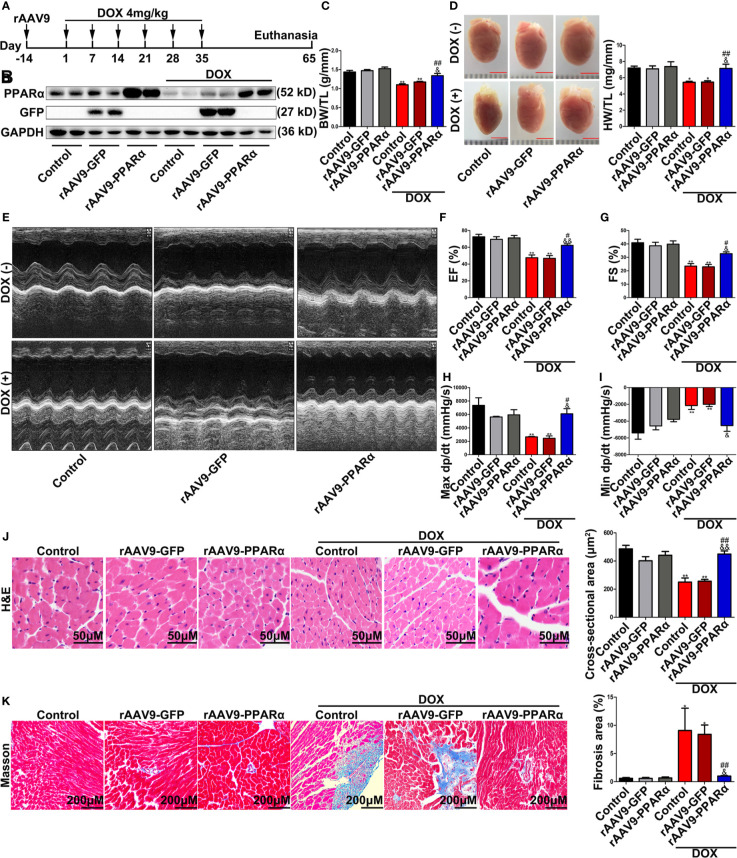
Overexpression of PPARα in the heart of DOX-induced mice improved cardiac function and reduced cardiotoxicity of DOX. **(A)** Schematic drawing of animal experiment 2. First, the mice were injected with rAAV9, and 2 weeks later, they were injected intraperitoneally with DOX (4 mg/kg) on the 1st, 7th, 14th, 21st, 28th, and 35th day, respectively. One month later, echocardiography was performed, and Millar catheter was inserted, followed by sacrifice. **(B)** Expression level of PPARα in heart tissues from DOX-induced mice was detected by Western blot, N = 2. **(C)** Body weight/tibia length ratio of mice in each group. N = 5–8. **(D)** Heart weight/tibia length ratio and corresponding representative pictures of mice in each group. N = 5–8, bar = 3 mm. **(E)** Representative echocardiography in M mode. **(F**, **G)** LVEF **(F)** and LVFS **(G)** of mice treated with DOX. N = 5–8. **(H**, **I)** Max dp/dt **(H)** and min dp/dt **(I)** of mice treated with DOX. N = 3. **(J)** Representative images and cross-sectional area of hearts from DOX-induced mice detected by H&E staining. N = 4–8, Bar = 50 μm. **(K)** Representative images of Masson’s trichrome staining and fibrosis area quantification. N = 4–8, Bar = 200 μm. *P < 0.05 vs. Control; **P < 0.01 vs. Control; ^#^P < 0.05 vs. DOX; ^##^P < 0.01 vs. DOX; ^&^P < 0.05 vs. DOX+rAAV9-GFP; ^&&^P < 0.01 vs. DOX+rAAV9-GFP.

Next, we overexpressed PPARα *in vivo* using the rAAV9 system. Western blot analyses showed that the expression levels of PPARα in mice administered rAAV9-PPARα were higher than those in control mice (**Figure 3B**). The DOX-treated mice obviously lost BW/TL and HW/TL compared with the control mice (**Figures 3C, D**). Moreover, cardiac function analyses showed that unlike in control mice, cardiac function was damaged in DOX-treated mice, as indicated by decreased left ventricular ejection fraction (LVEF), left ventricular fractional shortening (LVFS), max dp/dt, and min dp/dt (**Figures 3E–I**). H&E staining showed reduced cross-sectional area of the myocardium in DOX-treated mice (**Figure 3J**). Additionally, Masson′s trichrome staining revealed fibrosis in the heart of DOX-treated mice (**Figure 3K**). Remarkably, all these injuries were partially alleviated by of PPARα overexpression.

### PPARα Ameliorated Oxidative Stress Levels and Reduced Mitochondria-Dependent Apoptosis In Vivo and In Vitro

**Figure 4:**
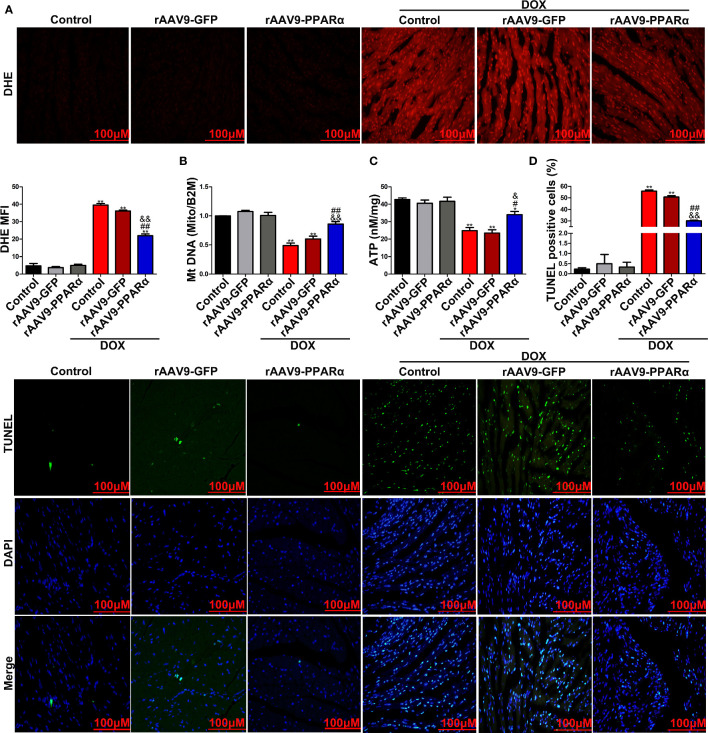
PPARα overexpression in the heart of DOX-induced mice improved mitochondria-dependent apoptosis. **(A)** Representative images and quantitative analysis of DHE staining in heart tissues of DOX-induced mice. N = 4, Bar = 100 μm. **(B)** MtDNA copy number of heart tissues from DOX-induced mice was detected by RT-PCR. N = 3. **(C)** Detection of ATP content in DOX-induced mice hearts by chemiluminescence assay. N = 3. **(D)** Representative images and quantitative analysis of TUNEL-FITC fluorescence staining in hearts of DOX-induced mice. N = 3, Bar = 100 μm. **P < 0.01 vs. Control; ^#^P < 0.05 vs. DOX; ^##^P < 0.01 vs. DOX; ^&^P < 0.05 vs. DOX+rAAV9-GFP; ^&&^P<0.01 vs. DOX+rAAV9-GFP.

DOX-induced apoptosis is a type of programmed cell death that is accompanied by activation of mitochondria-dependent signaling pathways. ROS is predominantly produced in the mitochondria, and thus, they frequently become the casualty of ROS exposure. Increased ROS levels can cause oxidative damage to the mtDNA, decrease mitochondrial transmembrane potential, and prevent ATP synthesis (Sinha et al., 2013). As shown in **Figure 4A**, DOX administration remarkably increased ROS generation, as determined by DHE staining, in DOX-induced murine hearts. However, overexpression of PPARα reversed the increase in DOX-induced ROS generation. Furthermore, DOX induction resulted in a significant decrease in mtDNA replication and ATP content; meanwhile, overexpression of PPARα abolished the destructive effects of DOX on mitochondrial function (**Figures 4B, C**). In addition, DOX significantly increased the percentage of TUNEL-positive cells, and the latter was reversed by overexpression of PPARα (**Figure 4D**). Collectively, these data suggest that mitochondria-dependent apoptosis in DOX-treated mice could be improved by overexpression of PPARα.

**Figure 5:**
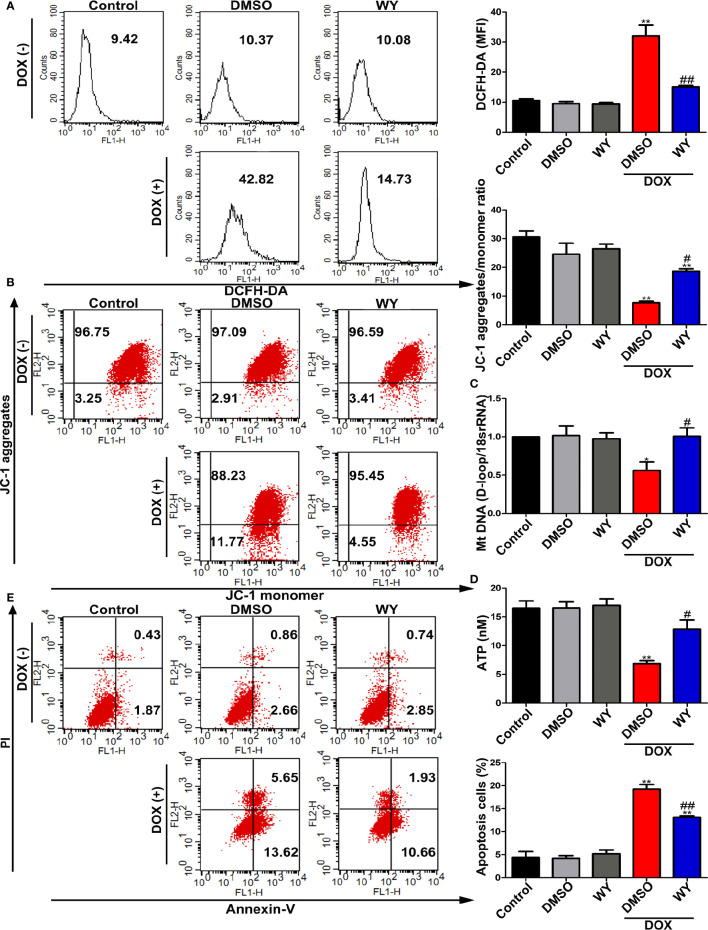
The activation of PPARα reduced mitochondria-dependent apoptosis in cardiomyocytes. **(A)** Intracellular ROS in H9C2 cells were quantitatively analyzed by flow cytometry. N = 4. **(B)** Representative images and quantitative analysis of flow cytometry for mitochondrial transmembrane potential in H9C2 cells by using fluorescent dye JC-1. N = 4. **(C)** MtDNA copy number in H9C2 cells was assessed by RT-PCR. N = 5. **(D)** ATP synthesis measured by chemiluminescence assay in H9C2 cells. N = 3. **(E)** Representative images and quantitative analysis of flow cytometry for apoptosis in H9C2 cells. N = 3. *P < 0.05 vs. Control; **P < 0.01 vs. Control; ^#^P < 0.05 vs. DOX; ^##^P < 0.01 vs. DOX.

We next verified the protective effects of PPARα on DOX-induced cardiotoxicity *in vitro*. As shown in **Figure 5A**, DOX treatment significantly elevated ROS level compared to those in the control group; this increase in ROS levels was inhibited by treatment with Wy-14643. To examine whether PPARα had a beneficial eﬀect on mitochondrial function, we assessed mitochondrial transmembrane potential, mtDNA copy number, and ATP content. As expected, DOX deteriorated mitochondrial function, as indicated by degressive mitochondrial transmembrane potential, mtDNA copy number, and ATP content, which were significantly ameliorated by Wy-14643 (**Figures 5B–D**). Consistently, pre-treatment with the PPARα-specific agonist, Wy-14643 significantly prevented DOX-induced cardiomyocyte apoptosis (**Figure 5E**). Together, these findings indicate that PPARα activation reduced ROS generation induced by DOX and improved mitochondrial function, thereby reducing mitochondria-dependent apoptosis.

### PPARα Directly Regulated the Expression of MEOX1

**Figure 6:**
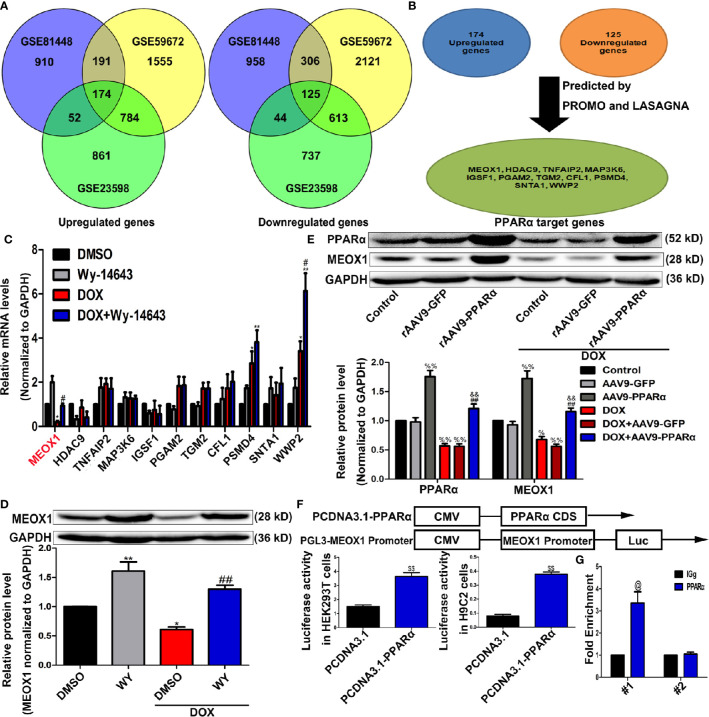
Identification of PPARα target genes. **(A)** Venn diagram showing the overlap for 174 upregulated genes and 125 downregulated genes from data sets GSE81448, GSE59672, and GSE23598. **(B)** PPARα target genes predicted by bioinformatics websites PROMO and LASAGNA. **(C)** Verification of bioinformatics results by RT-PCR in H9C2 cells. N = 3. **(D**, **E)** MEOX1 expression level measured by Western blotting analysis in H9C2 cells **(D)** and heart tissues **(E)**. N = 4. **(F)** Pattern diagram (top) of plasmid construction, and the regulation of MEOX1 *via* PPARα was determined by Dual luciferase assay in HEK293T cells (left of bottom) and H9C2 cells (right of bottom). **(G)** Direct regulation of PPARα on MEOX1 revealed by ChIP assay. N = 4 independent experiments. #1 and #2 represent site 1 and site 2, respectively. *P < 0.05 vs. DMSO; **P < 0.01 vs. DMSO; ^#^P < 0.05 vs. DOX; ^##^P < 0.01 vs. DOX; ^%^P < 0.05 vs. Control; ^%%^P<0.01 vs. Control; ^&&^P < 0.01 vs. DOX+rAAV9-GFP; ^$$^P < 0.01 vs. PCDNA3.1; ^@^P < 0.05 vs. IGg.

As a transcription factor, PPARα regulates the expression of many functional genes (Wilding, 2012; Pawlak et al., 2015). Therefore, we speculated that PPARα may play a protective role in DOX-induced cardiotoxicity by regulating the expression of downstream functional genes. We re-analyzed data sets GSE81448, GSE59672, and GSE23598, which were obtained from the hearts of control and DOX-treated mice to identify differentially expressed genes. The result of the intersectional analyses are represented in the form of a Venn diagram in **Figure 6A**; 174 upregulated and 125 downregulated genes were identified. To identify potential differentially expressed genes that may be regulated by PPARα, we used the online computational tools PROMO and LASAGNA. Finally, we identified 11 candidate genes (MEOX1, HDAC9, TNFAIP2, MAP3K6, IGSF1, PGAM2, TGM2, CFL1, PSMD4, SNTA1, and WWP2), as shown in **Figure 6B**. RT-PCR analysis showed that the mRNA expression level of MEOX1 was significantly downregulated following DOX treatment; however, the PPARα agonist Wy-14643 restored its levels (**Figure 6C**). The results of Western blot analysis *in vitro* were similar to those of RT-PCR (**Figure 6D**). In vivo, overexpression of PPARα reversed DOX-induced MEOX1 reduction (**Figure 6E**). Subsequently, we co-stained the heart sections of rAAV9-infected mice with anti-PPARα and anti-MEOX1. We found that the trend in MEOX1 expression in the hearts was consistent with that observed with PPARα treatment, which further validated our hypothesis (**Figure S3A**). To verify whether PPARα directly regulated the expression of MEOX1, we conducted luciferase reporter assay and CHIP assay. Luciferase activity of the MEOX1 reporter was significantly increased by pcDNA3.1-PPARα compared to that observed after transfection of an empty vector plasmid in HEK293T or H9C2 cells (**Figure 6F**). The result of the CHIP assay showed that PPARα could bind directly to a specific region (−1734 to −1724 bp) of the MEOX1 promoter in HEK293T cells (**Figure 6G**). These results suggest that PPARα may exert biological effects by directly regulating the expression of MEOX1.

### PPARα-Mediated Amelioration in ROS Production and Mitochondria-Dependent Cardiomyocyte Apoptosis Depended on MEOX1

**Figure 7:**
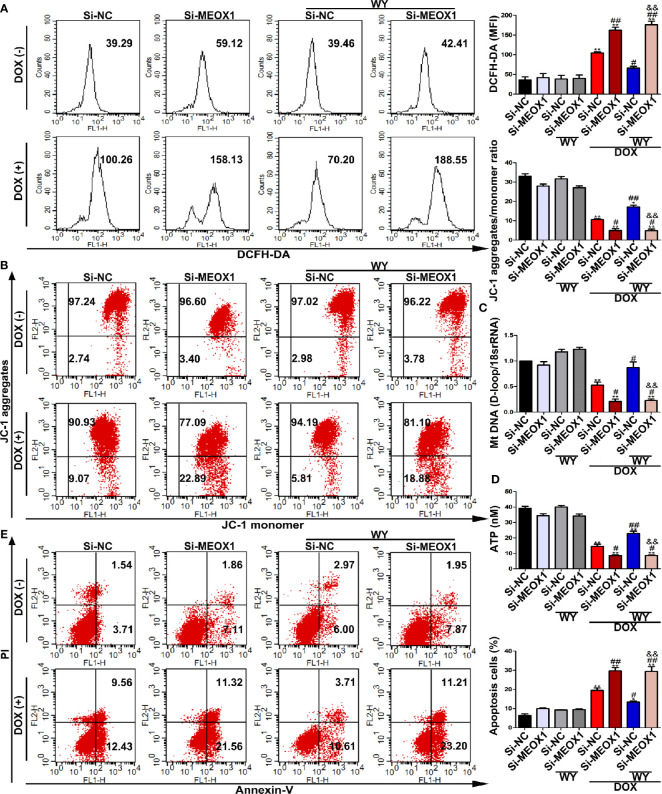
The protective effect of PPARα activation in DOX-induced cardiomyocyte was neutralized by knocking down MEOX1. **(A)** Intracellular reactive oxygen species detected by flow cytometry in H9C2 cells. N = 3. **(B)** Representative images and quantitative analysis for mitochondrial transmembrane potential in H9C2 cells measured by flow cytometry. N = 3. **(C)** Quantification of mtDNA copy number in H9C2 cells was performed by RT-PCR. N = 3. **(D)** ATP content in H9C2 cells was detected by bioluminescent assay. N = 3. **(E)** DOX-induced apoptosis in H9C2 cells was assessed by flow cytometry. N = 3. *P < 0.05 vs. Si-NC; **P < 0.01 vs. Si-NC; ^#^P < 0.05 vs. Si-NC+DOX; ^##^P < 0.01 vs. Si-NC+DOX; ^&&^P < 0.01 vs. Si-NC+DOX+Wy-14643.

To verify whether PPARα has a protective effect by directly regulating MEOX1, we knocked down MEOX1 before using PPARα agonists, Wy-14643, and DOX. As expected, Wy-14643 significantly decreased ROS levels induced by DOX; however, the protective effect of Wy-14643 was cancelled out when Si-MEOX1 was applied (**Figure 7A**). Interestingly, this counteraction was not only observed at the ROS levels, but also showed a similar phenomenon on mitochondrial transmembrane potential, mtDNA copy number, and ATP content (**Figures 7B–D**). Furthermore, the beneficial effect of Wy-14643 on cardiomyocyte apoptosis was reversed by Si-MEOX1 (**Figure 7E**). Overall, these results reveal that PPARα alleviated the mitochondria-dependent cardiomyocyte apoptosis by directly regulating MEOX1.

## Discussion

**Figure 8:**
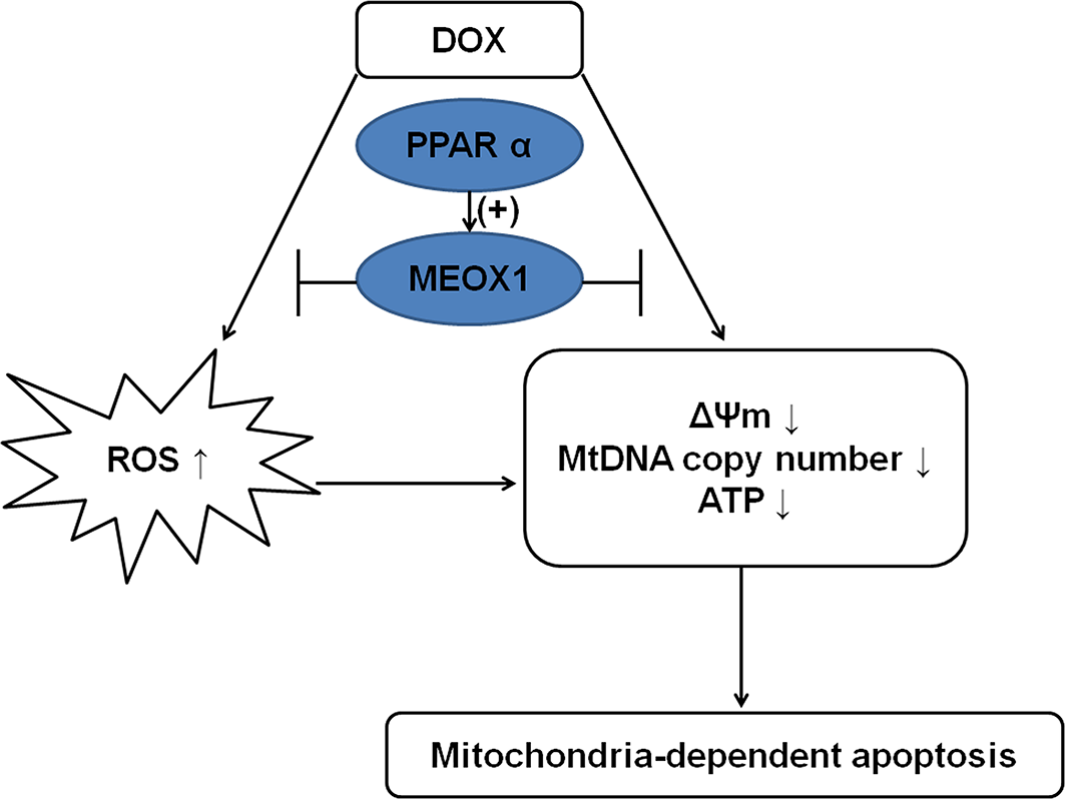
Mechanism diagram showing the protective effect of PPARα on DOX-induced cardiotoxicity. PPARα promoted MEOX1 transcription, leading to inhibition of ROS production and improvement in mitochondrial function, which decreased mitochondria-dependent apoptosis.

The present study show that PPARα can upregulate the expression of MEOX1, thereby ameliorating mitochondrial function deteriorated by DOX, and finally reducing mitochondria-dependent apoptosis (**Figure 8**). These findings suggest that MEOX1 might be an additional protective target of PPARα. Furthermore, these data revealed an innovative pathway through which PPARα can affect mitochondrial function, thereby providing a novel therapeutic strategy against DOX-induced cardiotoxicity.

Although DOX is effective against many types of cancer and plays an irreplaceable role in tumor chemotherapy, it causes serious side effects, which can also be fatal. An increasing number of cancer survivors are at risk of cardiotoxicity caused by anthracycline drugs (more than 5 million people in the United States alone) (Brown et al., 2015). Thus, DOX-induced cardiotoxicity has a significant negative effect on the long-term survival and quality of life of patients undergoing chemotherapy. DOX-induced chronic cardiomyopathy is dose-dependent, and patients may develop dilated cardiomyopathy accompanied by cardiac dysfunction after several years of DOX treatment, which may finally lead to heart failure and death. To date, many studies on DOX-induced cardiotoxicity are ongoing for decades. The pathogenesis of DOX-induced cardiotoxicity is very complex, and these mechanisms eventually lead to cell death (Zhang et al., 2009; Vejpongsa and Yeh, 2014; Renu et al., 2018). Dexrazoxane is the only drug approved by Food and Drug Administration (FDA) to prevent DOX-induced cardiotoxicity. It was initially considered that dexrazoxane can protect the myocardium from DOX-induced damage by chelating iron in the cells, and by reducing the levels of oxidative stress; however, it was later found that dexrazoxane can interact with TOP2β, thus preventing the binding of DOX to the latter (Zhang et al., 2012; Wenningmann et al., 2019). Of note, the DOX-TOP2β complex inhibits the expression of PPARα by binding to the gene promoter of PPARα (Brown et al., 2015). Moreover, the ROS produced by DOX may also contribute to the down-regulation of PPARα, leading to a subsequent series of adverse effects (Robinson and Grieve, 2009). Our results showed that the expression of PPARα in DOX-treated cardiomyocytes decreased, both *in vivo* and *in vitro*, but there was no similar change in tumor tissues. Interestingly, weakened cardiomyocyte viability and increased cardiomyocyte apoptosis were observed. Therefore, we paid attention to the relationship between PPARα and cardiomyocyte apoptosis induced by DOX.

As known, topoisomerase is necessary for DNA replication, recombination, and transcription (Champoux, 2001). DOX is capable of binding to topoisomerase 2α (TOP2α) and TOP2β. Once DOX binds to them, they can form a complex that interferes with the transcriptome and cause cell death *via* various pathways. TOP2α is abundantly expressed in the tumor tissues; therefore, a combination of DOX and TOP2α is the molecular basis for eliminating tumor cells. Unfortunately, the expression of TOP2β is abundant in the heart, and the binding of DOX to TOP2β is related to cardiotoxicity (Zhang et al., 2012; Wenningmann et al., 2019). However, role of PPARα in DOX chemotherapy is unclear. Thus, we next tested the combined effect of DOX and FENO on tumors *in vitro* and *in vivo*. The effect of PPARα on tumor growth is not entirely clear, and PPARα plays different roles in various types of tumors. As previously reported, Zhang et al. showed that, in the case of hypoglycemia and hypoxia, CD8-positive T-cells activated the PPARα pathway and enhanced the ability of the lipid metabolism to enhance the killing effect of the PD-1 blockade on the melanoma (Zhang et al., 2017). In addition, FENO inhibited the proliferation of lung cancer cells by inhibiting the activation of NF-kB and ERK pathways, and played a synergistic role with budesonide in TP53 wild type A549 cells (Liang et al., 2014). The effect of PPARα activation is the opposite in the liver cancer, PPARα activation enhanced the expression of MYC, which in turn enhanced the expression of the Krt23, a downstream target gene of the PPARα, thereby promoted the proliferation of the liver cancer cells (Kim et al., 2019). In this study, three different types of tumor cells were used to test the effect of PPARα activation on their ability of growth. Our data showed that the activation of PPARα does not promote the growth of these three tumor cells at least, and, interestingly, PPARα activation can improve cardiac dysfunction induced by DOX. PPARα has a wide range of biological effects, usually known as its role in regulating lipid metabolism (Vosper et al., 2002). FENO, a commercial drug of PPARα agonist, is usually used in the treatment of hyperlipidemia for its role in reducing TG and LDL and increasing HDL. In addition, PPARα also has the effect of immunomodulation and vasodilation. The activation of PPARα is also considered to be beneficial to atherosclerosis, myocardial ischemia-reperfusion and hypertension (Diep et al., 2004; Bulhak et al., 2006; Ichihara et al., 2006; Tordjman et al., 2007; Pawlak et al., 2015). Herein, we used rAAV9 to manipulate the high expression of PPARα in the heart and found that high expression of PPARα reduced DOX-induced cardiotoxicity and improved cardiac function, which suggested that PPARα is also of positive significance in antagonising DOX-induced cardiotoxicity.

The pathogenic mechanisms of DOX-induced cardiotoxicity appear to be diverse, involving lipid peroxidation, oxidative stress, DNA/RNA damage, autophagy, mitochondrial dysfunction, inflammation, apoptosis, and calcium homeostasis disorder. Importantly, elevated levels of oxidative stress is a particularly major issue that leads to cardiotoxicity, as this can be explained by the chemical structure of DOX (Zhang et al., 2009). The structural and functional integrity of mitochondria is critical to the physiological function of the cardiovascular system. Moreover, the mitochondria-dependent signal pathway is a particularly powerful pathway that regulates apoptosis (Outomuro et al., 2007; Dominic et al., 2014). Usually, the mitochondria are the sites where most of the ROS are produced. Once the cumulative dose of DOX exceeds 500 mg/m^2^, the levels of oxidative stress increase. Moreover, a small amount of DOX can produce a large amount of ROS through multiple redox reactions. DOX is transformed to semiquinone in the mitochondria by ROS-producing enzymes *via* single-electron reduction of quinone moiety, followed by transformation into a ring C. Semiquinones can react with oxygen to produce superoxide anions (O_2_-), which can generate toxic and highly reactive hydroxyl radicals (OH∙) during the Fenton reaction, finally resulting in ROS generation. The resulting ROS can react with nearby mitochondrial biomolecules, including proteins, lipids, and nucleic acids. DOX can also react with mtDNA, forming a complex that interferes with normal mitochondrial function, protein expression, and lipid oxidation (Cappetta et al., 2017; Koleini et al., 2019; Songbo et al., 2019). In this experiment, the level of oxidative stress elevated by DOX was inhibited by PPARα, *in vivo* and *in vitro*, whereas the levels of oxidative stress increased further after knocking down MEOX1, but this increased trend could not be reversed by PPARα activation, suggesting that the reduction of oxidative stress levels by PPARα was likely *via* MEOX1.

It is well known that mitochondria are the main cell organelles associated with ROS production; however, they are also the subject to the negative effects of ROS, which lead to a fatal outcome. Some toxic reagents, such as DOX, can induce the opening of mitochondrial permeability transition pore (MPTP), resulting in a decrease in mitochondrial transmembrane potential, increase in mitochondrial membrane permeability, and release of apoptotic proteins from the mitochondria. Meanwhile, due to the oxidative damage of mtDNA, synthesis of mitochondrial ATP is inhibited, which leads to mitochondria-dependent apoptosis (Monteiro et al., 2013; Sinha et al., 2013; Sterba et al., 2013). Therefore, we considered whether PPARα had any effect on mitochondrial dysfunction induced by DOX. Interestingly, both *in vitro* and *in vivo*, the damage cause by DOX to the mitochondria was partially recovered by PPARα and apoptosis was eventually reduced. However, when MEOX1 was knocked down, the protective effects of PPARα were not observed. In other words, the existence of MEOX1 is crucial for the function of PPARα against DOX-induced cardiotoxicity.

Our research also has certain limitations: we could not determine how MEOX1 regulates mitochondrial function, and this needs to be confirmed by future studies. In addition, MEOX1 was previously shown to cause pressure overload-induced hypertrophy (Lu et al., 2018). We assumed that under normal physiological conditions, MEOX1 expression is maintained at certain levels. Overexpression or decreased expression of MEOX1 in cardiomyocytes may alter physiological conditions that transform into pathological conditions. However, this is also a hypothesis that requires further validation. Hence, we would like to emphasize that MEOX1 may play different roles in different pathological situations *via* different mechanisms. Taken together, we have proved that PPARα can improve mitochondrial function and reduce DOX-induced cardiotoxicity by regulating the expression of MEOX1.

In conclusion, our findings suggest that PPARα may be a potential target against DOX-induced cardiotoxicity.

## Data Availability Statement

All datasets generated for this study are included in the article/**Supplementary Material**.

## Ethics Statement

The animal study was reviewed and approved by Experimental Animal Research Committee of Tongji Medical College, Huazhong University of Science and Technology, Wuhan, China.

## Author Contributions

LW originally designed the scientific research. WW performed experiments and data analyses and wrote the manuscript. YW monitored the project progression. ZZ, QF, and DW provided technical assistance. WW, QF, LW, and YW were responsible for revision of this manuscript. All authors contributed to the article and approved the submitted version.

## Funding

This study was supported by foundations from National Natural Science Foundation of China (grants 81790624, 81570308, and 81900244).

## Conflict of Interest

The authors declare that the research was conducted in the absence of any commercial or financial relationships that could be construed as a potential conflict of interest.

## Abbreviations

DAPI, 4′,6-diamidino-2-phenylindole; DCFH-DA, 2′,7′-dichlorofluorescein diacetate; JC-1, 5,5′,6,6′-Tetrachloro-1,1′,3,3′-tetraethylbenzimidazolylcarbocyanine iodide; BW/TL, body weight/tibia length ratio; rAAV9, recombinant adeno-associated virus serotype 9; CCK8, cell counting kit-8; DHE, dihydroethidium; DOX, doxorubicin; DMEM, Dulbecco’s modified Eagle’s medium; EGFP, enhanced green fluorescent protein; FENO, fenofibrate; FDA, Food and Drug Administration; H&E, hematoxylin and eosin; HW/TL, heart weight/tibia length ratio; LVEF, left ventricular ejection fraction; LVFS, left ventricular fractional shortening; MEOX1, mesenchyme homeobox 1; mtDNA, mitochondrial DNA; MPTP, mitochondrial permeability transition pore; ΔΨm, mitochondrial transmembrane potential; PPARα, peroxisome proliferation activated receptor α; RT-PCR, real-time polymerase chain reaction; ROS, reactive oxygen species; RXR, retinoic acid receptor; siRNA, small-interfering RNA; TUNEL, terminal deoxynucleotidyl transferase (TdT)-mediated dUTP-biotin nick end-labeling; TOP2α, topoisomerase 2α; TOP2β, topoisomerase 2β; WY, Wy-14643.
