# Supplementary material for: PPARα Ameliorates Doxorubicin-Induced Cardiotoxicity by Reducing Mitochondria-Dependent Apoptosis via Regulating MEOX1
Source: Front Pharmacol. 2020 Oct 8;11:528267. doi: 10.3389/fphar.2020.528267 (PMC7578427; doi:10.3389/fphar.2020.528267)
Supplement: Supplementary file 1 [file DataSheet_1.doc]

**Supplementary Figure 1**
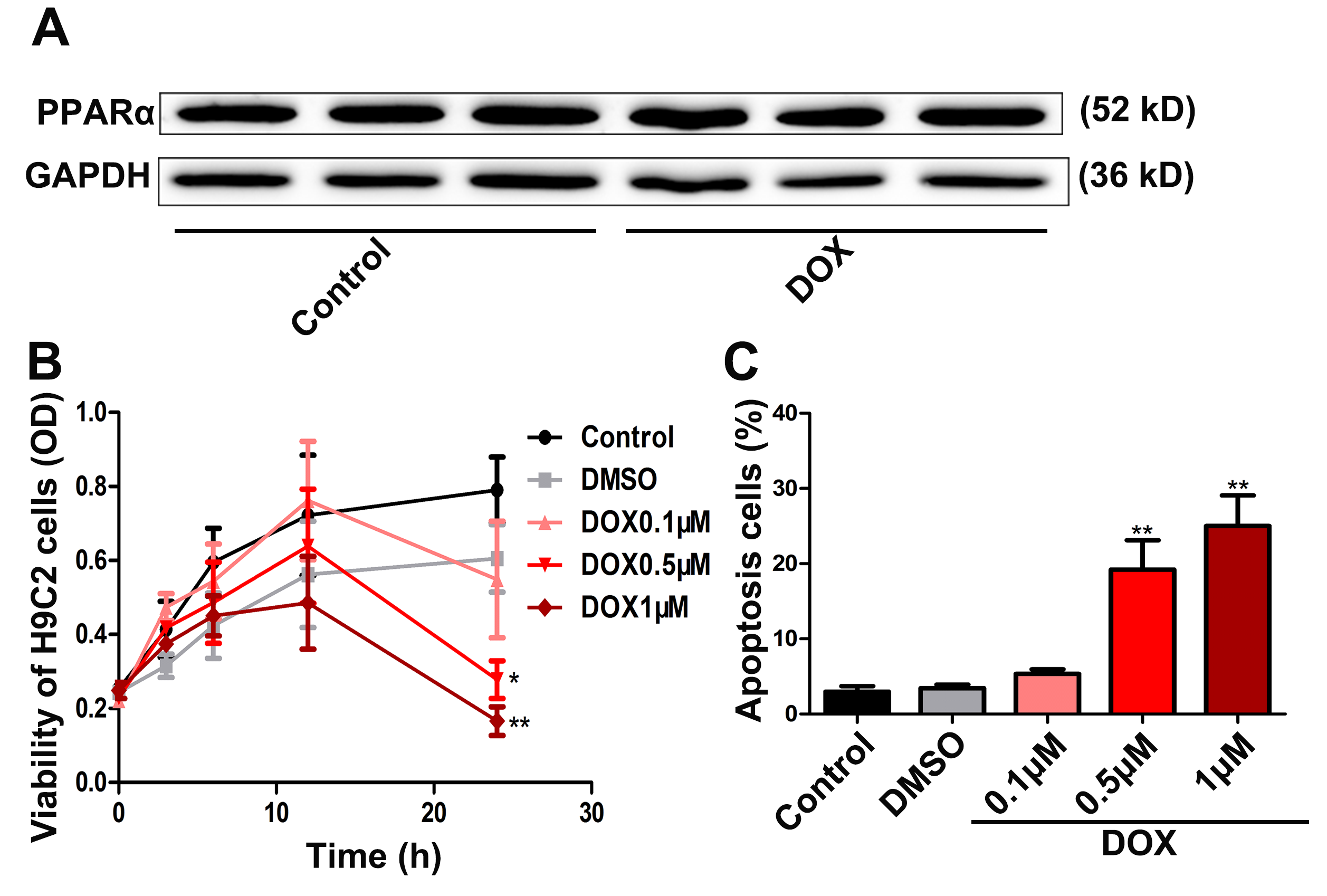


**Figure S1.** A, Detection of PPARα expression in tumour tissues after DOX treatment by western blot, N=3. B.  Viability of H9C2 cells reduced by DOX. N=3. C, Quantitative analysis of flow cytometry for apoptosis in H9C2 cells treated with different concentrations of DOX. N=3. *P<0.05 vs. Control; **P<0.01 vs. Control.

**Supplementary Figure 2**

**
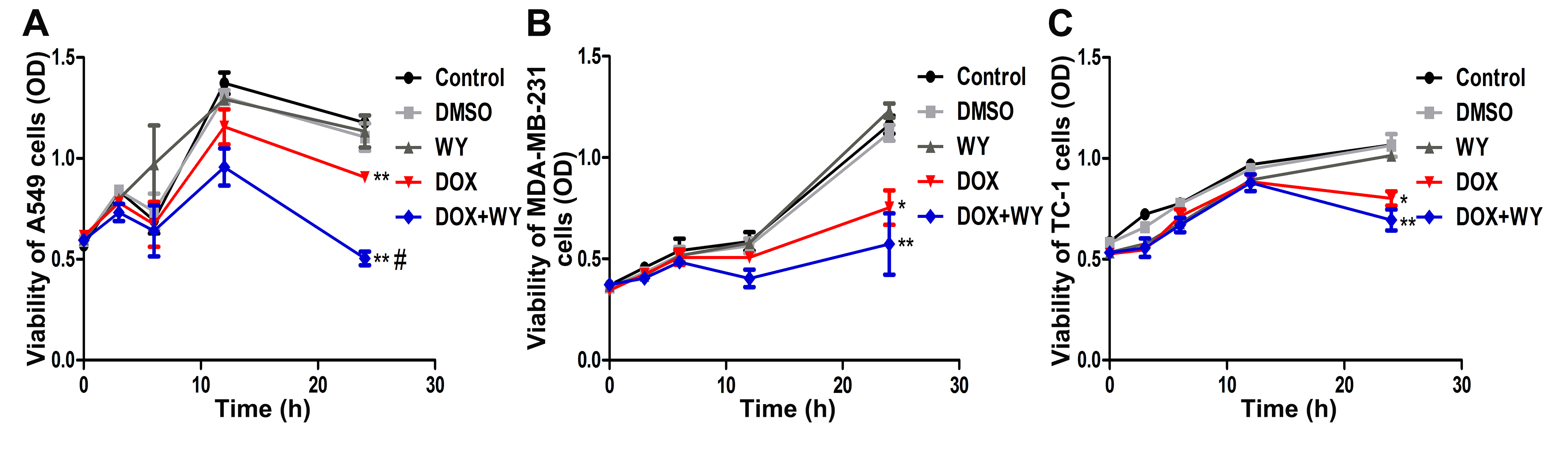
**

**Figure S2.** A-C, Viability of A549 (A), MDA-MB-231 (B), and TC-1 cells (C) treated with DOX with or without Wy-14643. N=3. *P<0.05 vs. Control; **P<0.01 vs. Control; ^#^P<0.05 vs. DOX; ^##^P<0.01 vs. DOX.

**Supplementary Figure 3**

**
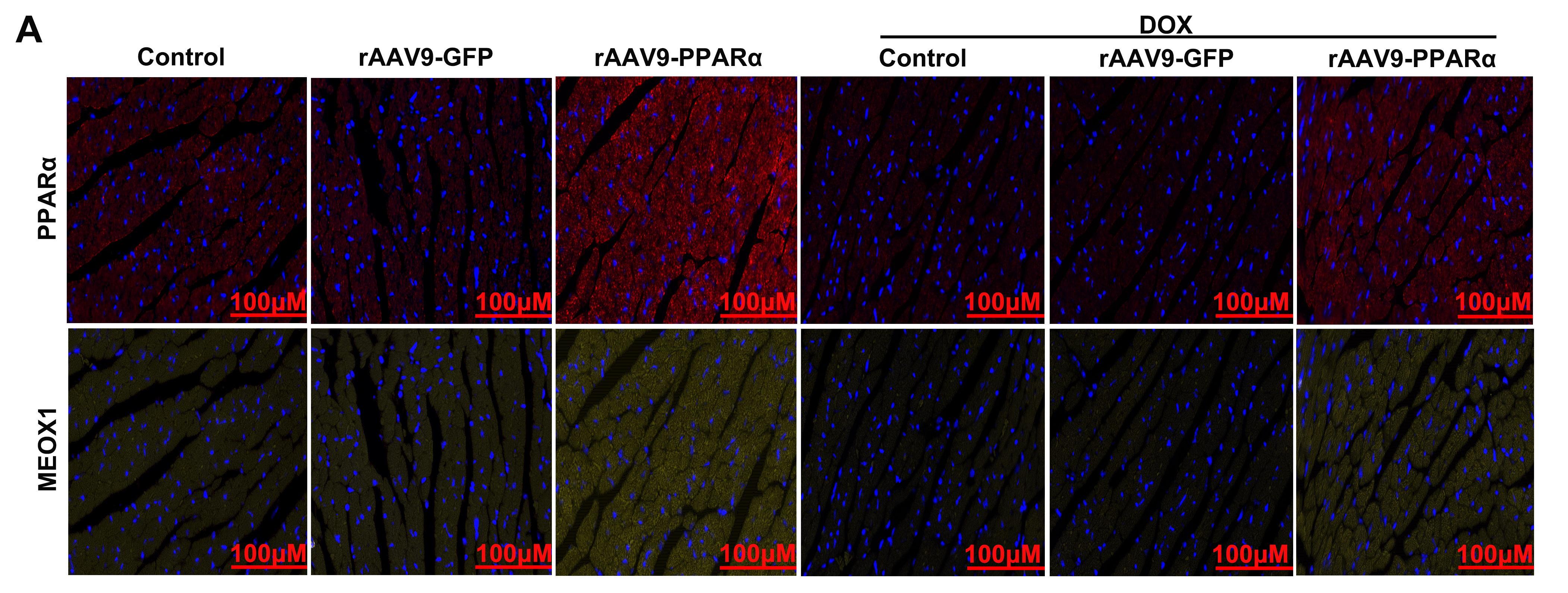
**

**Figure S3.** A, The representative images of co-staining with anti-PPARα and anti-MEOX1 on the sections of the hearts infected by rAAV9.
