# Supplementary figures and images for: PPARα Ameliorates Doxorubicin-Induced Cardiotoxicity by Reducing Mitochondria-Dependent Apoptosis via Regulating MEOX1
Source: Front Pharmacol. 2020 Oct 8;11:528267. doi: 10.3389/fphar.2020.528267 (PMC7578427; doi:10.3389/fphar.2020.528267)

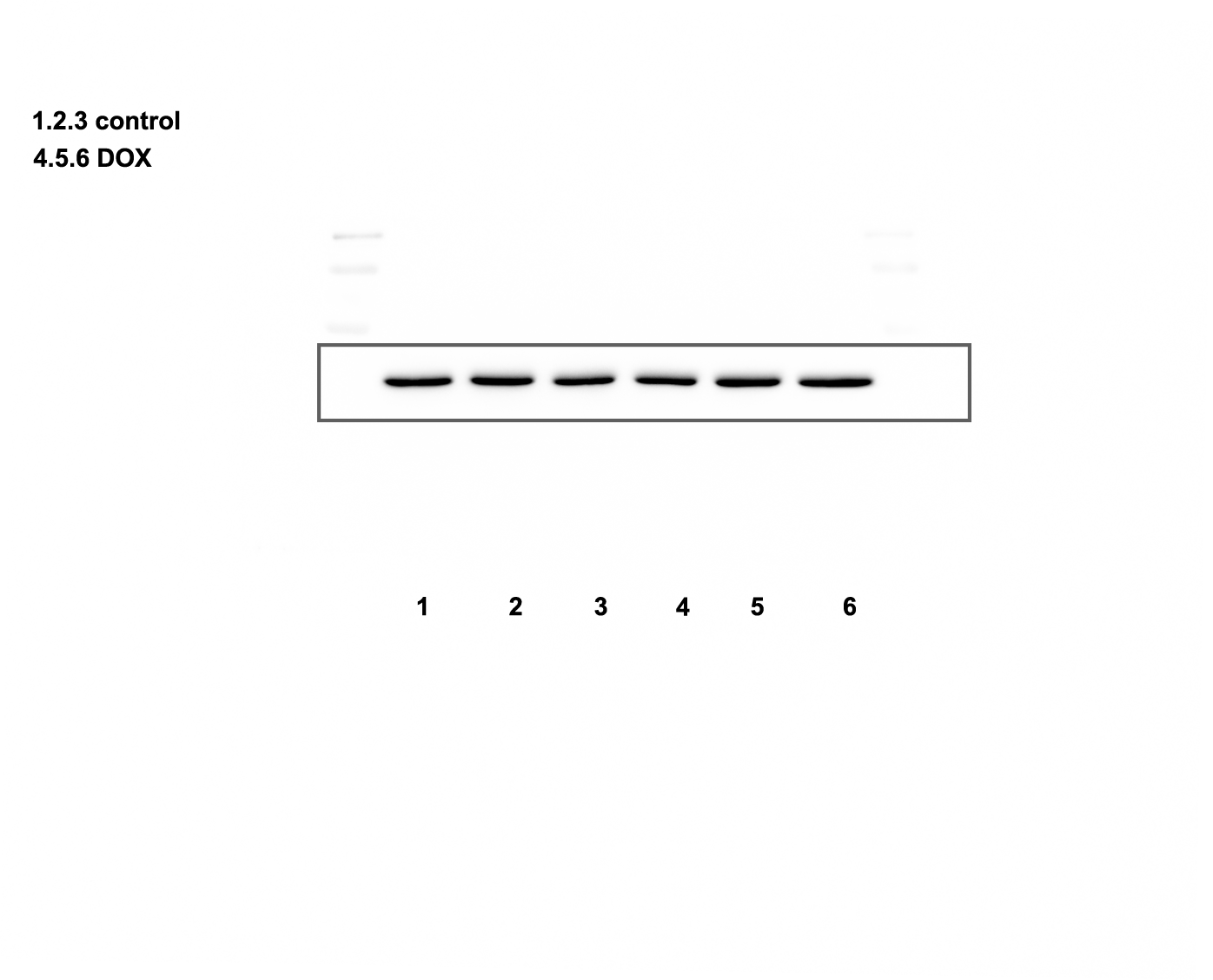

Supplement: Supplementary file 2 [file DataSheet_2.zip › original image files/Figure1A-GAPDH.tif]

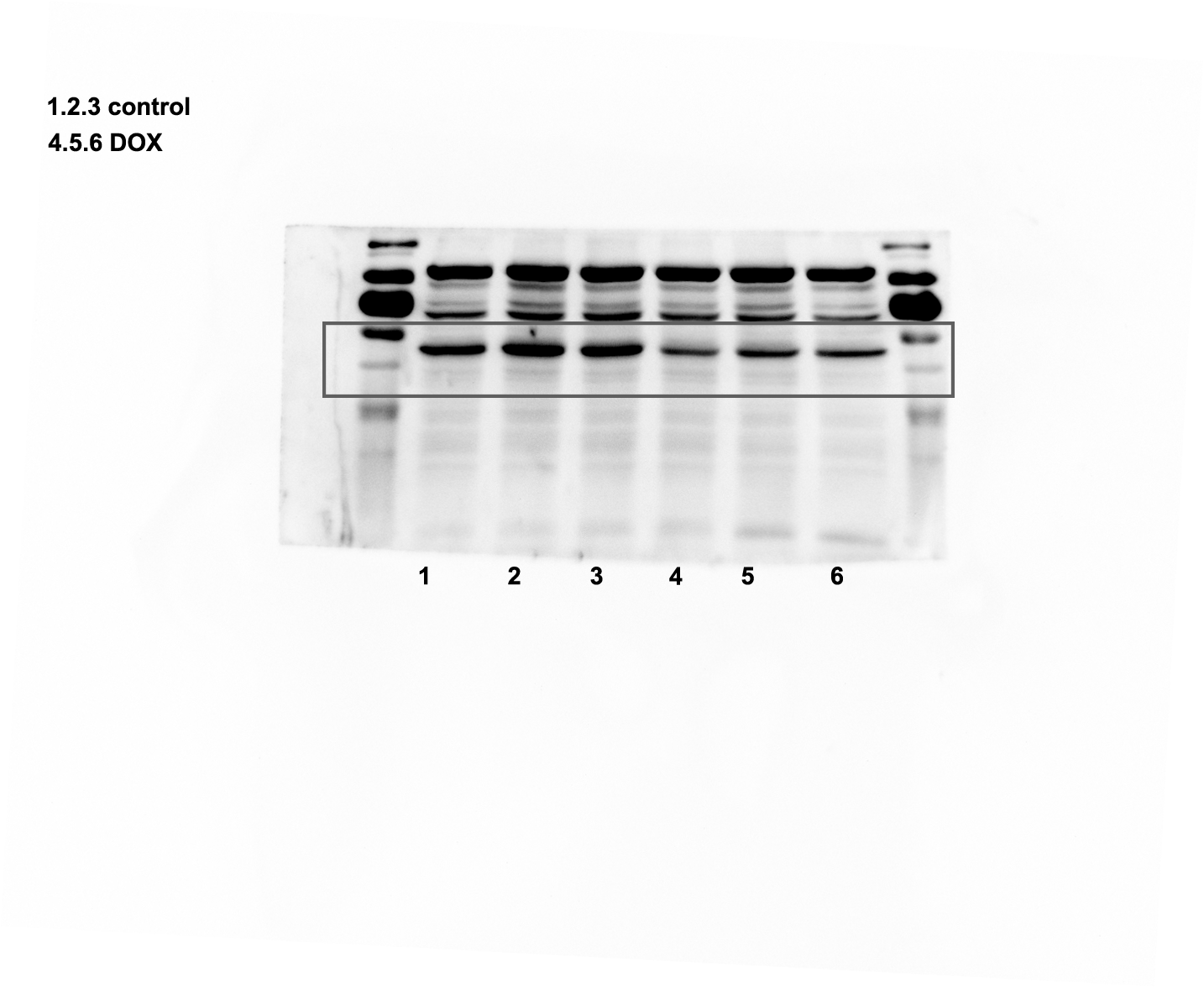

Supplement: Supplementary file 2 [file DataSheet_2.zip › original image files/Figure1A-PPARA.tif]

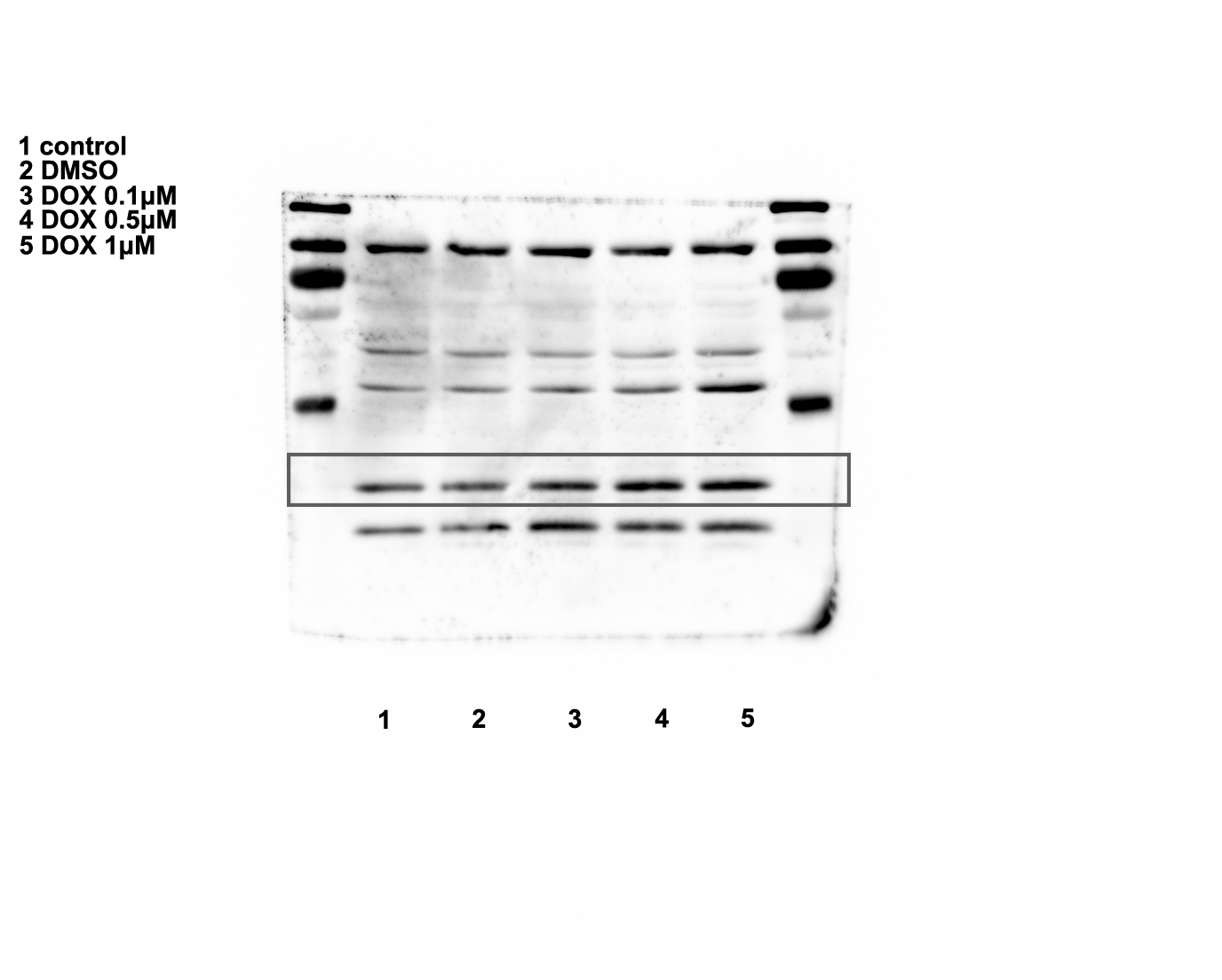

Supplement: Supplementary file 2 [file DataSheet_2.zip › original image files/Figure1B-BAX.tif]

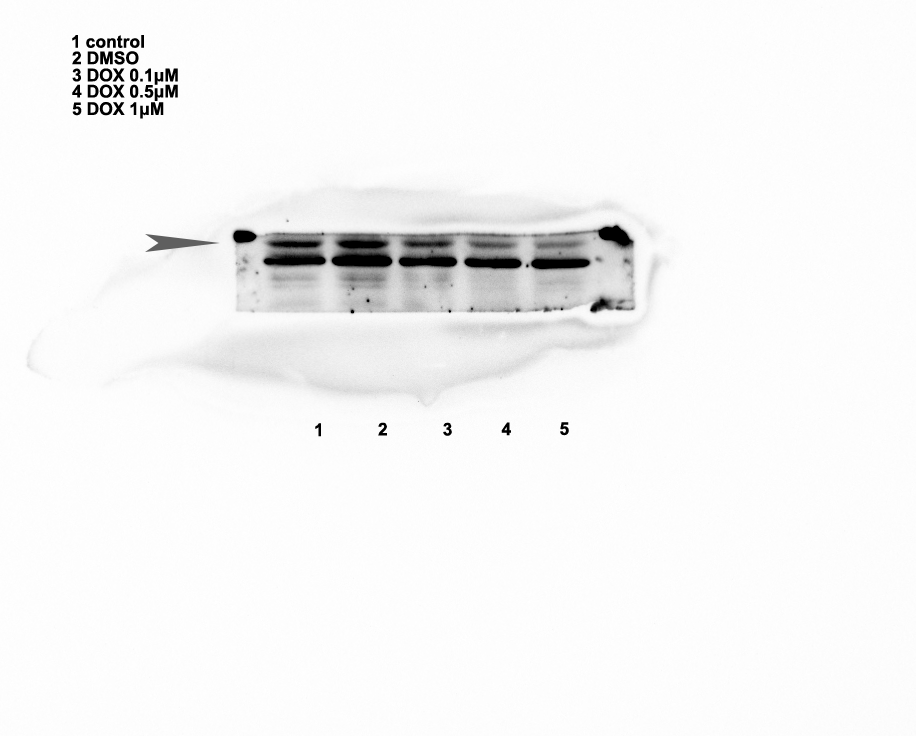

Supplement: Supplementary file 2 [file DataSheet_2.zip › original image files/Figure1B-BCL2.tif]

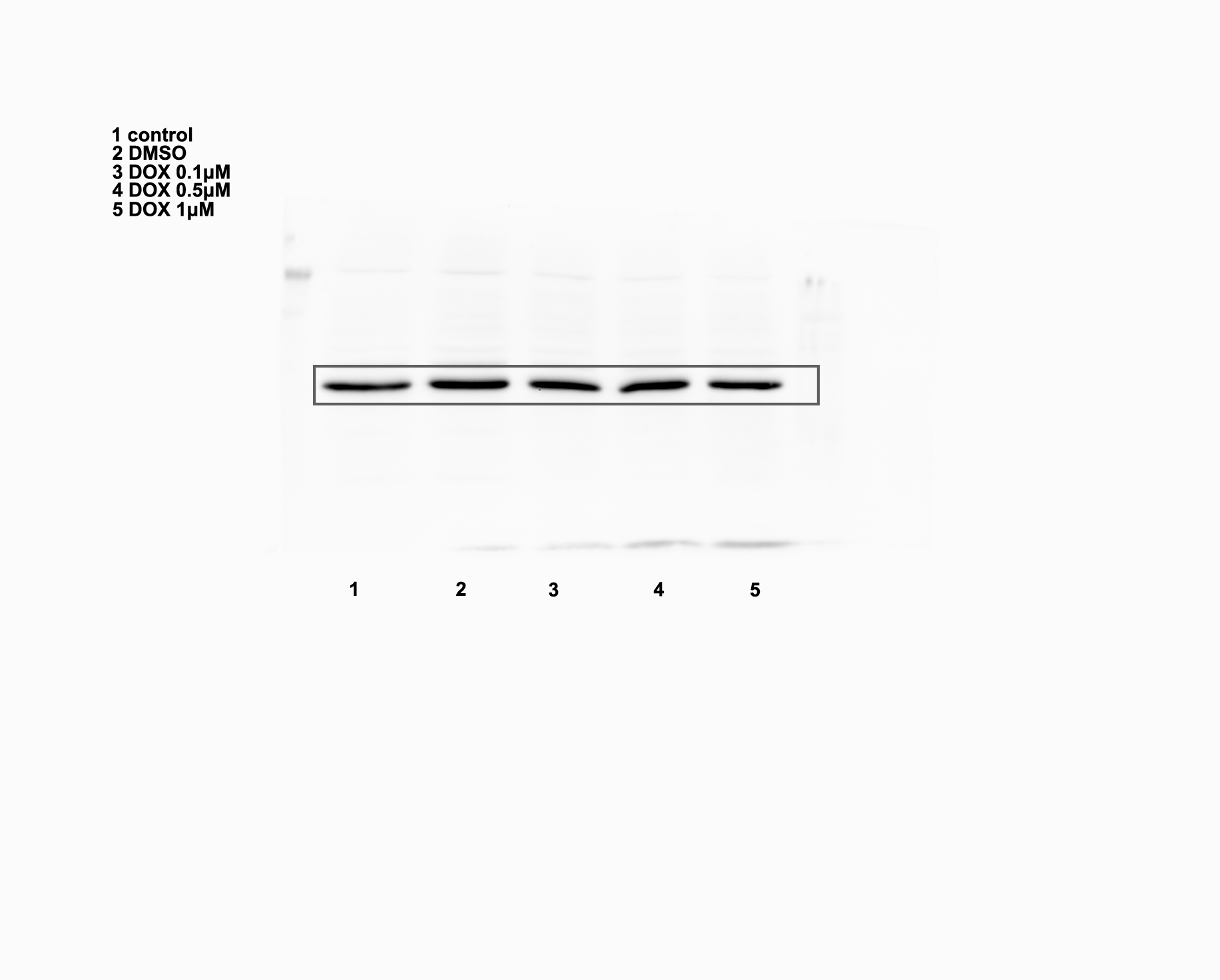

Supplement: Supplementary file 2 [file DataSheet_2.zip › original image files/Figure1B-GAPDH.tif]

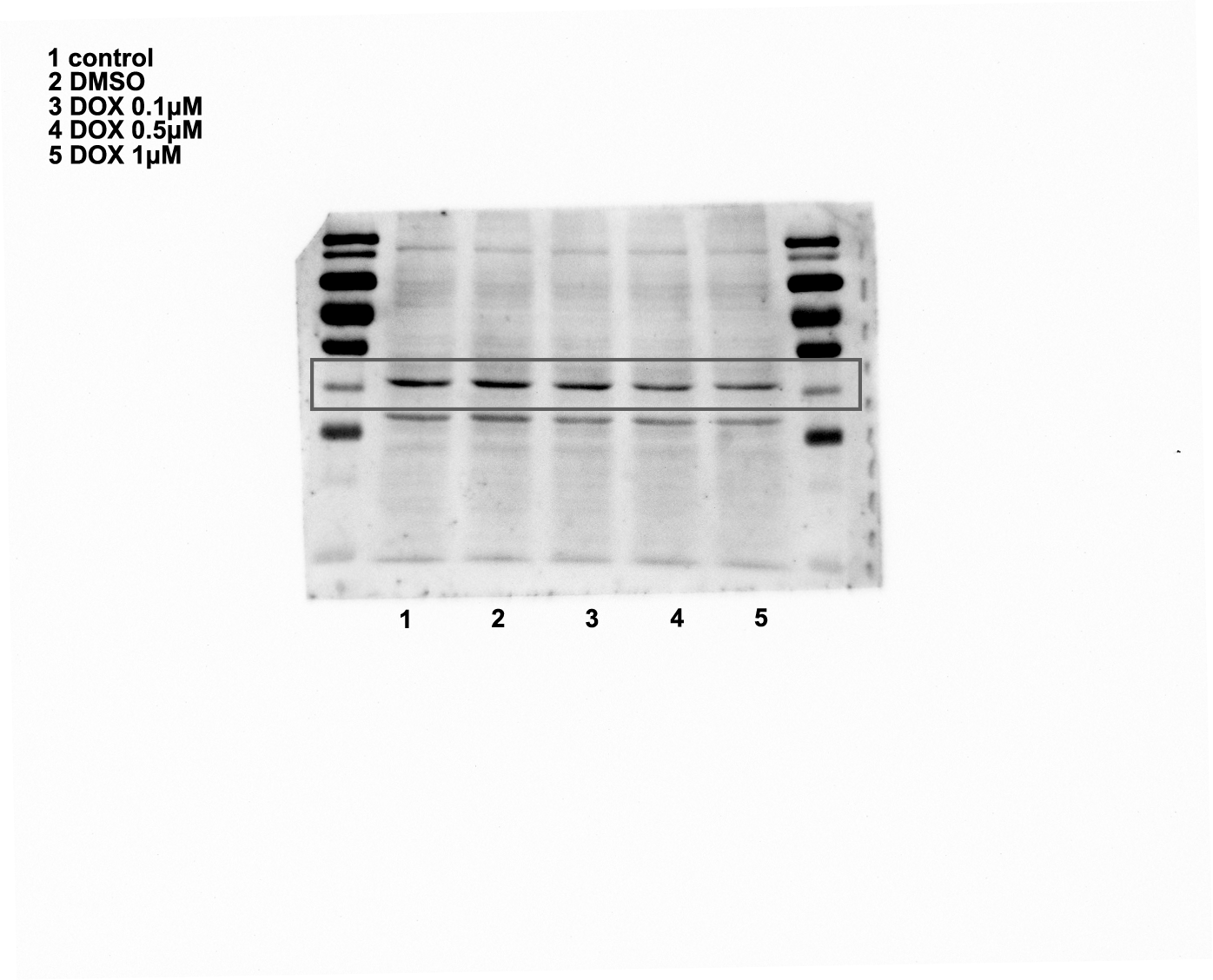

Supplement: Supplementary file 2 [file DataSheet_2.zip › original image files/Figure1B-PPARA.tif]

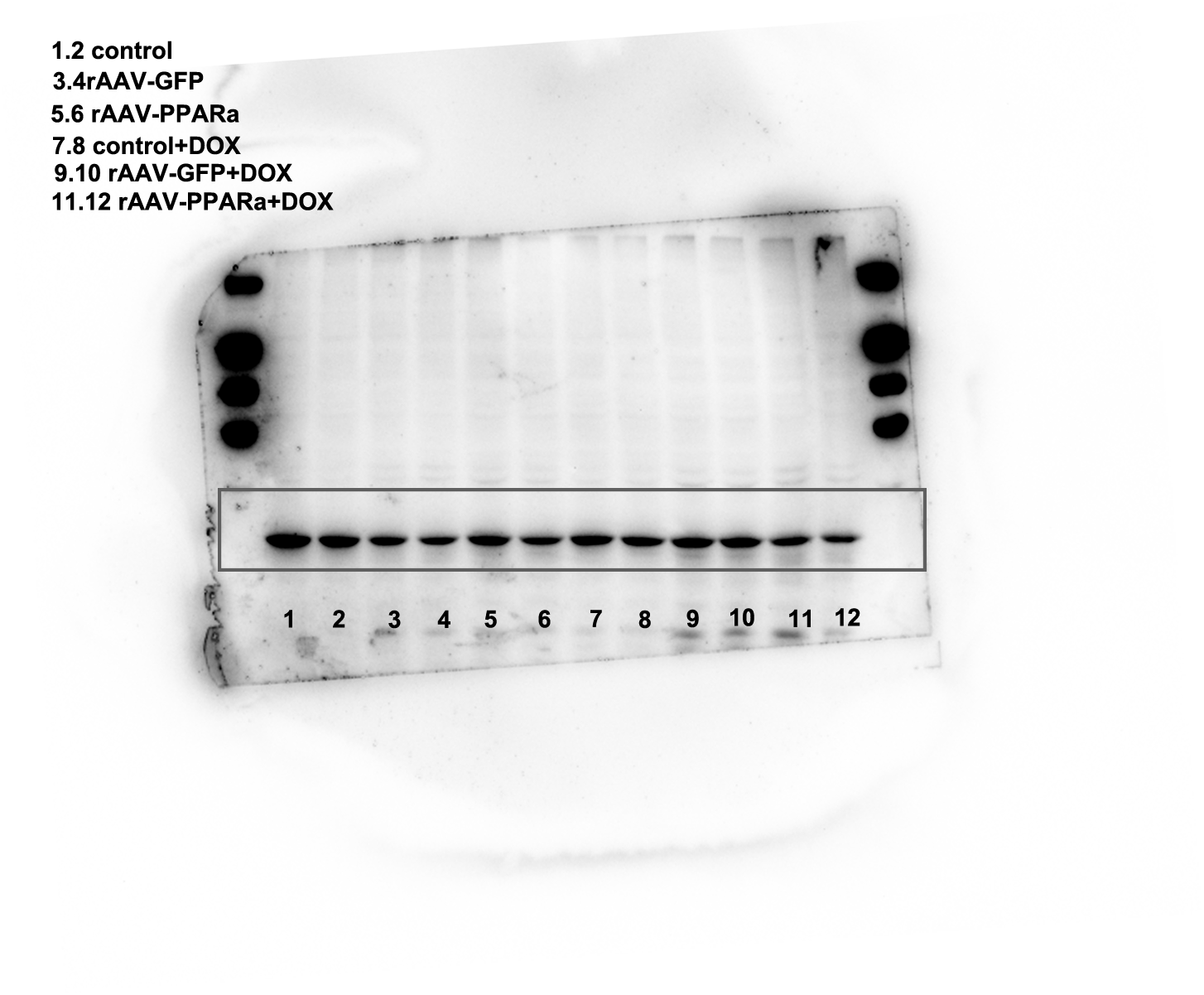

Supplement: Supplementary file 2 [file DataSheet_2.zip › original image files/Figure3B-GAPDH.Tif]

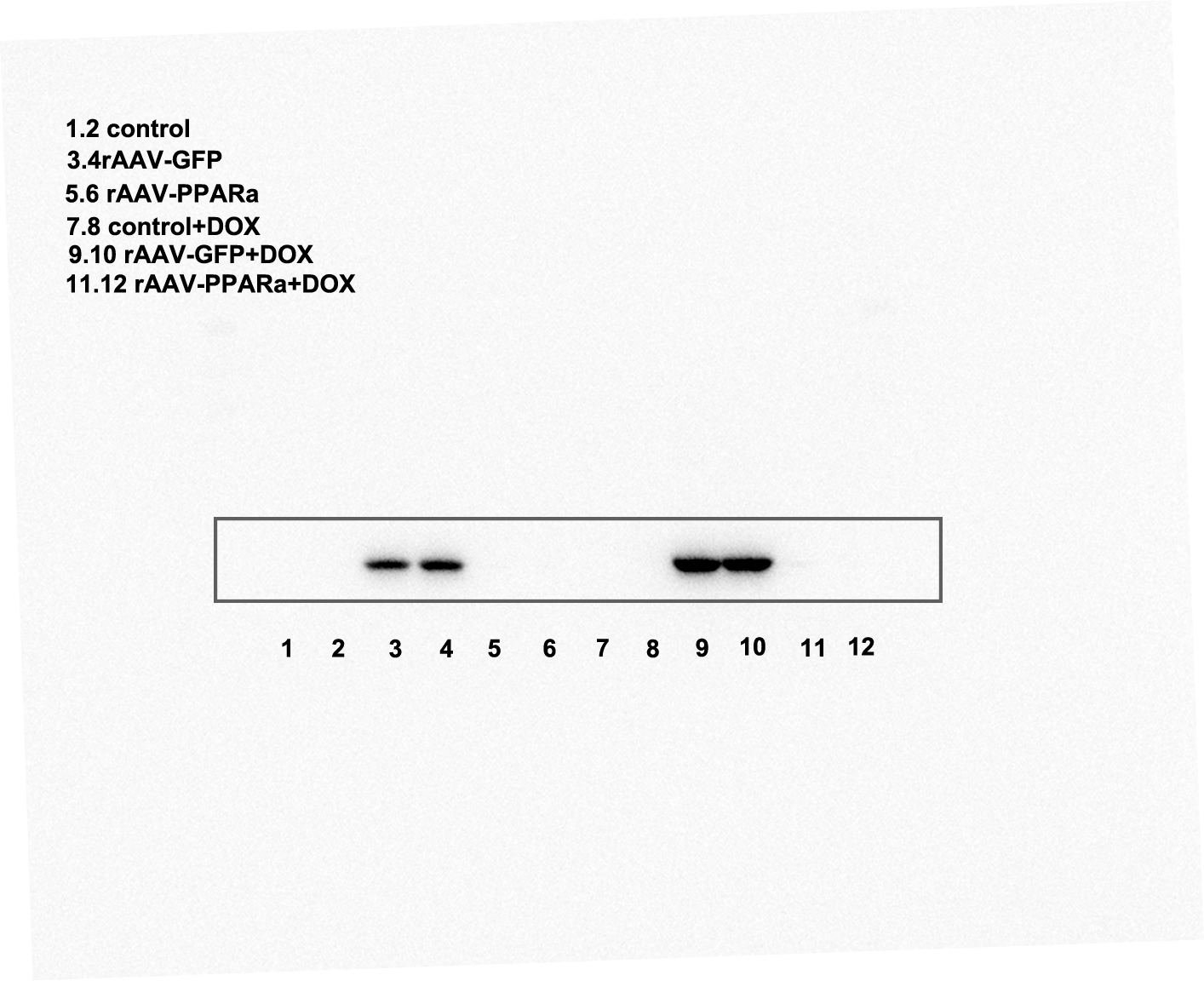

Supplement: Supplementary file 2 [file DataSheet_2.zip › original image files/Figure3B-GFP.Tif]

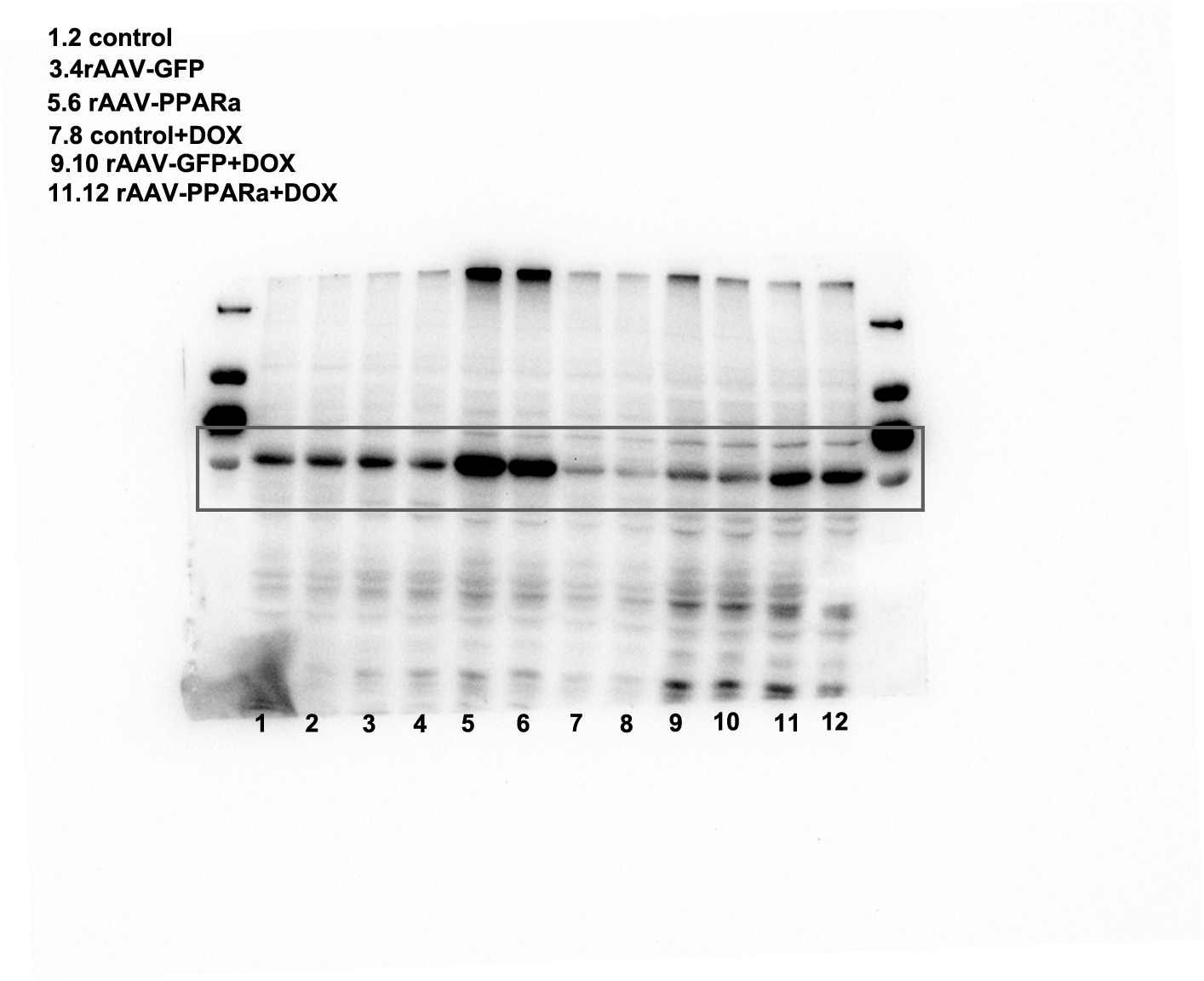

Supplement: Supplementary file 2 [file DataSheet_2.zip › original image files/Figure3B-PPARA.Tif]

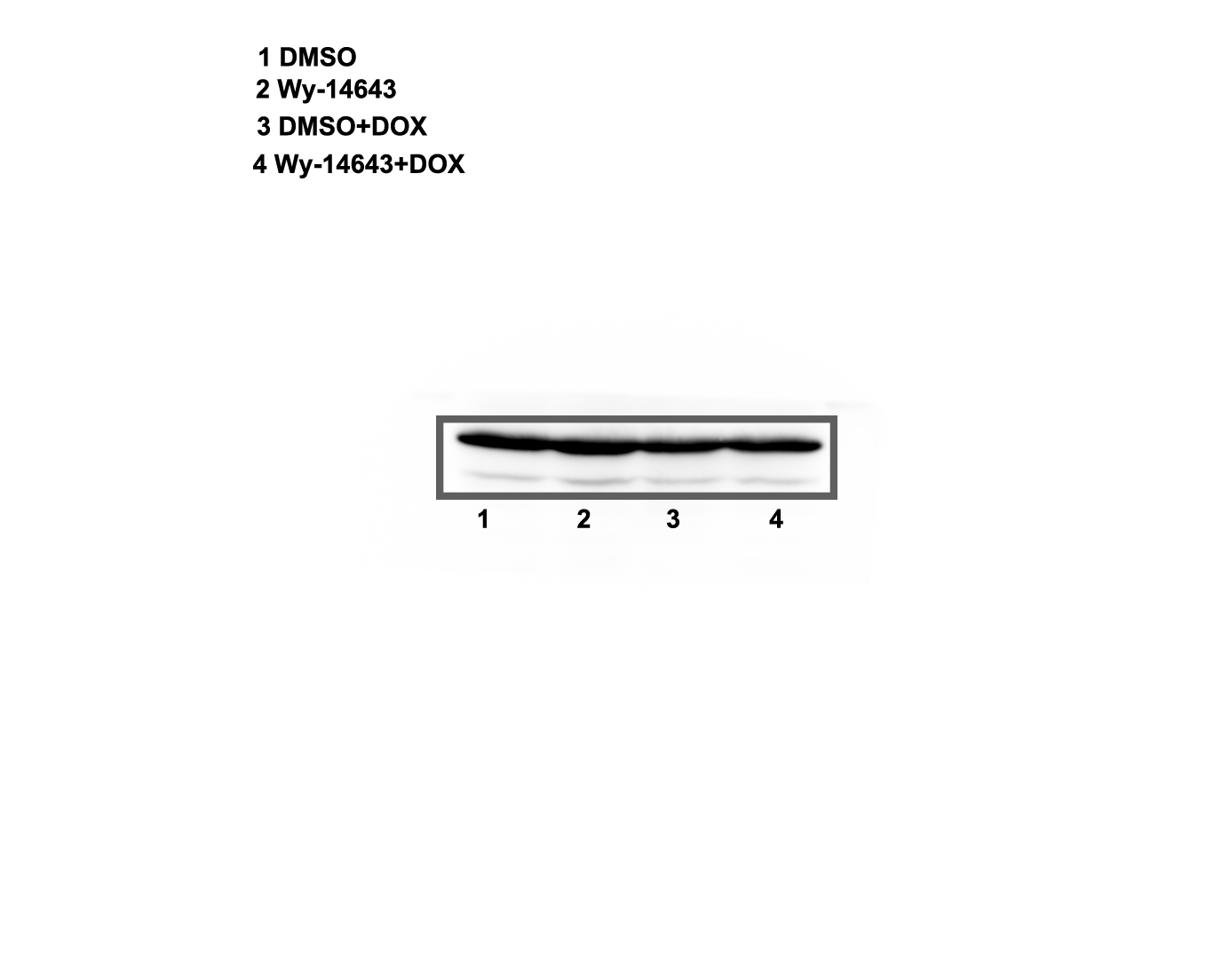

Supplement: Supplementary file 2 [file DataSheet_2.zip › original image files/Figure6D-GAPDH.tif]

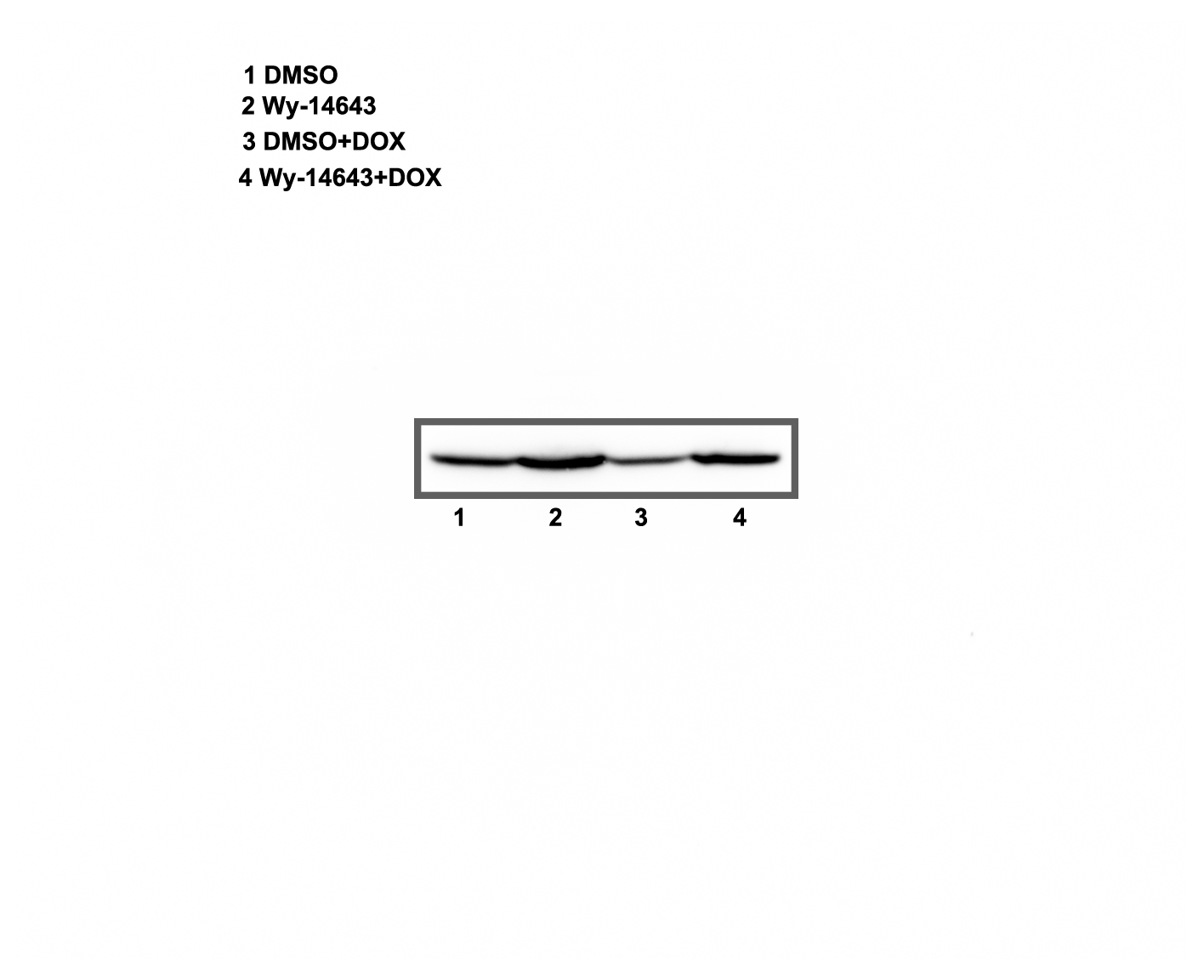

Supplement: Supplementary file 2 [file DataSheet_2.zip › original image files/Figure6D-MEOX1.tif]

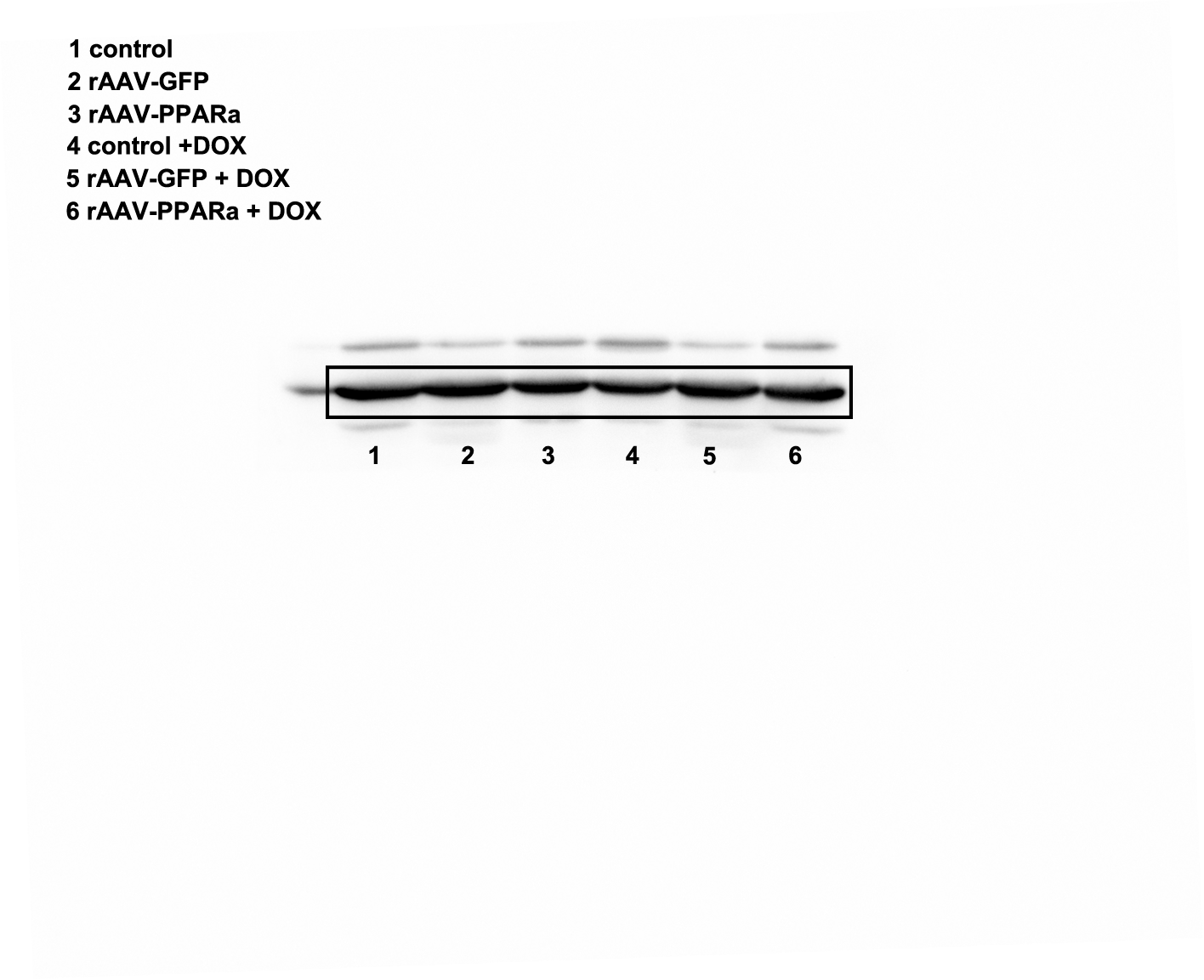

Supplement: Supplementary file 2 [file DataSheet_2.zip › original image files/Figure6E-GAPDH.tif]

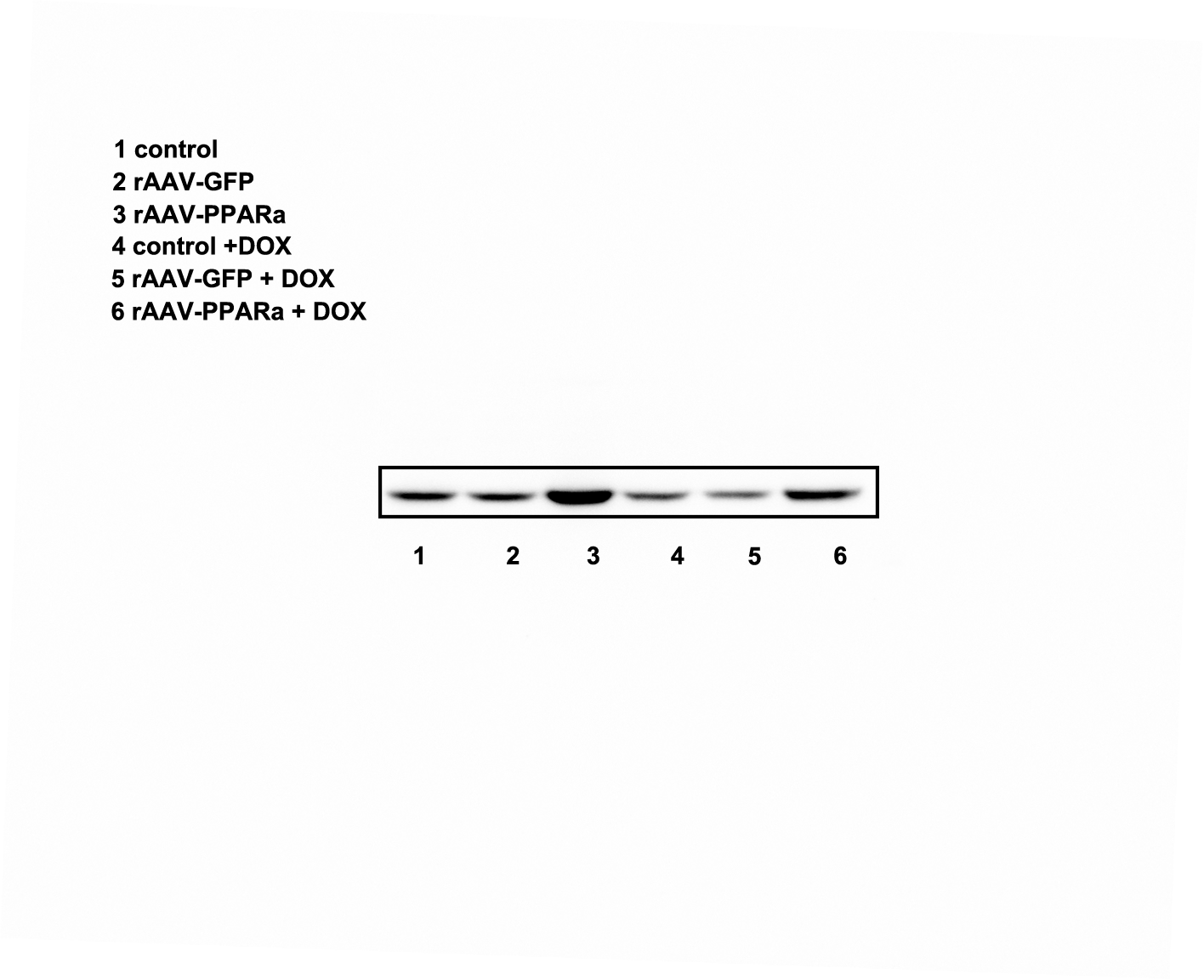

Supplement: Supplementary file 2 [file DataSheet_2.zip › original image files/Figure6E-MEOX1.tif]

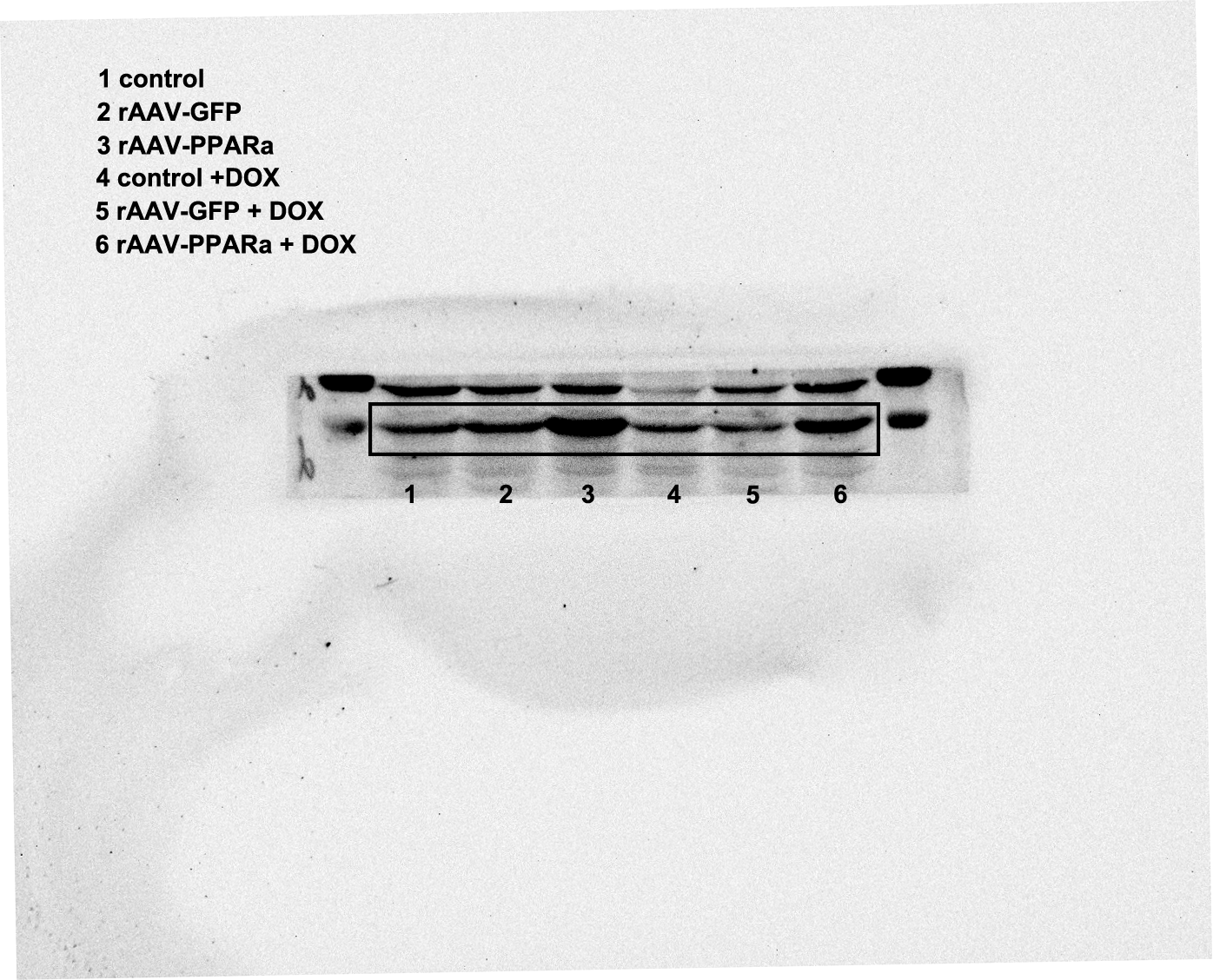

Supplement: Supplementary file 2 [file DataSheet_2.zip › original image files/Figure6E-PPARA.tif]

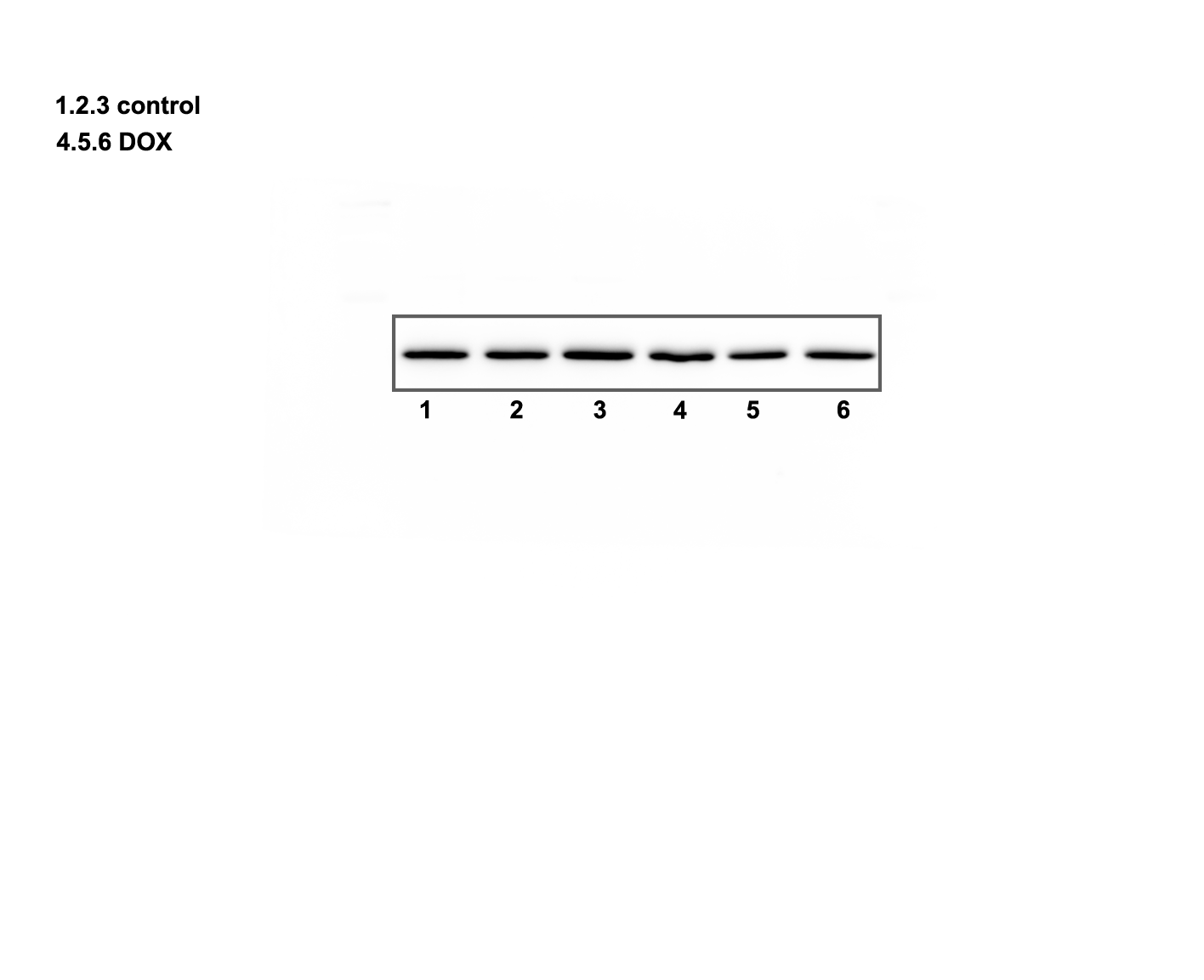

Supplement: Supplementary file 2 [file DataSheet_2.zip › original image files/Supplementary Figure1A-GAPDH.tif]

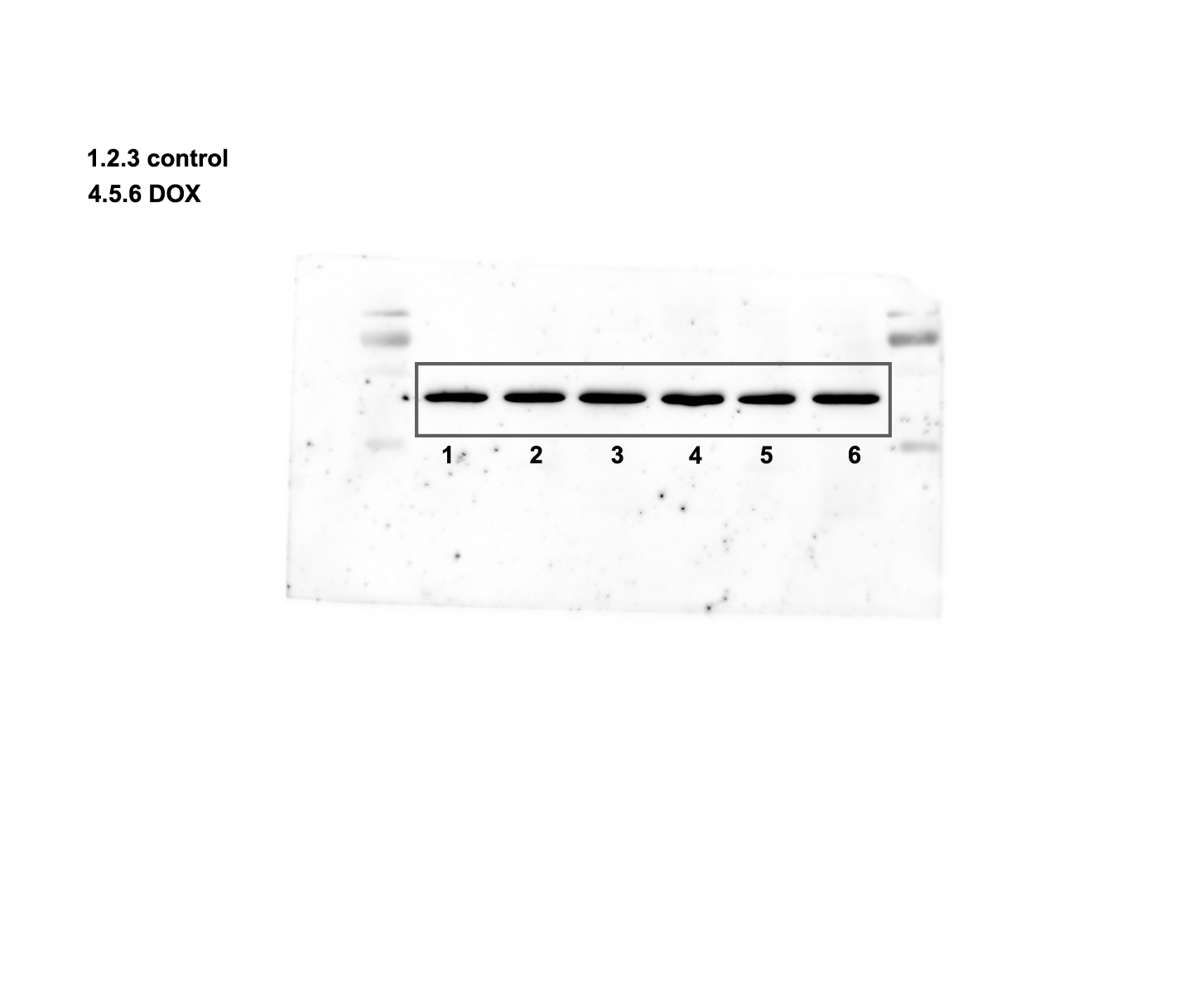

Supplement: Supplementary file 2 [file DataSheet_2.zip › original image files/Supplementary Figure1A-PPARA.tif]
