# Supplementary material for: PPARα Ameliorates Doxorubicin-Induced Cardiotoxicity by Reducing Mitochondria-Dependent Apoptosis via Regulating MEOX1
Source: Front Pharmacol. 2020 Oct 8;11:528267. doi: 10.3389/fphar.2020.528267 (PMC7578427; doi:10.3389/fphar.2020.528267)
Supplement: Supplementary file 3 [file Table_1.docx]

**Supplementary Table 1. List of primary antibodies for Western blot.**

| **Antibody** | **Catalogue number#** | **Brand** |
| --- | --- | --- |
| anti-BAX | ab32503 | Abcam (UK) |
| anti-BCL2 | ab196495 | Abcam (UK) |
| anti-PPARα | ab24509 | Abcam (UK) |
| anti-GAPDH | ab8245 | Abcam (UK) |
| anti-MEOX1 | ab105349 | Abcam (UK) |
| anti-GFP | BM3883 | Boster (China) |

**Supplementary Table 2. List of primers for RT-PCR.**

| **Gene** | **Forward** | **Reverse** |
| --- | --- | --- |
| PPARα | GGCAGAAATCCTTACCTGTGAAC | TTCCTGCGAGTATGACCCG |
| MEOX1 | AGAGAATGGAGGAGGAAAGCC | CAGGTAGTTATGGTGGGCGAA |
| HDAC9 | CCCCAGCATCCTGTACATTTC | GCCTTCTCCCAGACCTACTCC |
| TNFAIP2 | GAACACTTCGCCACCCACC | GCTCCCAAGCCTGATACCCT |
| MAP3K6 | TGACAGGCTTCGGGACATTC | TCAGGAGCGTTTGGATAGTGG |
| IGSF1 | GGGTTGCCATGCTCGTCA | CCCAGTCCCGTCCTTTAGTAG |
| PGAM2 | GCTATCCGCACCCTTTGG | TCTTCACCTGCTCCTCCCC |
| TGM2 | AAGTATGATGCGTCCTTCGTGT | TTAGTGCTGATCTTCTGCCCC |
| CFL1 | TGCTCTCTATGACGCAACCTATG | TTGTAATTCGTGCTTGATTCCTG |
| PSMD4 | CCGAAGCAACCCTGAGAATAAC | TCTTGCCTTTGGGCTGGAC |
| SNTA1 | GGCATCAGCATCAAGGGAG | CATCGCCGACAAAGAGGG |
| WWP2 | GGACGCAGGGAATGATACAGG | GTGGCAGAGGAAGCGGAACT |
| GAPDH | TTCCTACCCCCAATGTATCCG | CATGAGGTCCACCACCCTGTT |
| β-actin | CATCCTGCGTCTGGACCTGG | TAATGTCACGCACGATTTCC |

**Supplementary Table 3.** **Echocardiography analyses of tumor-bearing mice treated by DOX.**

|  | **Control** | **FENO** | **DOX** | **DOX+FENO** |
| --- | --- | --- | --- | --- |
| LVAW-d (mm) | 0.6807±0.08992 | 0.6590±0.06546 | 0.5364±0.03228 | 0.5602±0.02940 |
| LVAW-s (mm) | 1.167±0.1196 | 1.089±0.07112 | 0.9167±0.05207 | 1.100±0.05049 |
| LVID-d (mm) | 3.209±0.1623 | 2.872±0.1834 | 3.707±0.1720 | 3.319±0.1963 |
| LVID-s (mm) | 1.777±0.1575 | 1.559±0.1158 | 2.546±0.1151** | 1.867±0.1262^##^ |
| LVPW-d (mm) | 0.7365±0.04761 | 0.7945±0.1026 | 0.7435±0.05968 | 0.5980±0.03684 |
| LVPW-s (mm) | 1.229±0.09336 | 1.175±0.06651 | 0.9642±0.05943* | 1.010±0.03552 |
| LVEF (%) | 80.92±2.550 | 82.78±1.952 | 59.40±3.559** | 82.47±0.6673^##^ |
| LVFS (%) | 48.81±2.679 | 50.32±2.186 | 31.10±2.235** | 50.20±0.8034^##^ |

*P<0.05 vs. Control; **P<0.01 vs. Control; ^#^P<0.05 vs. DOX; ^##^P<0.01 vs. DOX.

**Supplementary Table 4.** **Data of hemodynamic analyses of tumor-bearing mice treated by DOX.**

|  | **Control** | **FENO** | **DOX** | **DOX+FENO** |
| --- | --- | --- | --- | --- |
| Max dp/dt (mmHg/s) | 5756±511.2 | 7945±1090 | 2479±384.1* | 5170±290.8^#^ |
| Min dp/dt (mmHg/s) | -5263±806.1 | -7295±928.4 | -2561±452.8* | -4754±95.03 |

*P<0.05 vs. Control; **P<0.01 vs. Control; ^#^P<0.05 vs. DOX; ^##^P<0.01 vs. DOX.

**Supplementary Table 5. Echocardiography data of mice injected with adeno-associated virus.**

|  | **Control** | **rAAV9-GFP** | **rAAV9-****PPAR****α** | **DOX** | **DOX+rAAV9-GFP** | **DOX+rAAV9-PPARα** |
| --- | --- | --- | --- | --- | --- | --- |
| LVAW-d (mm) | 0.6515±0.07766 | 0.6778±0.06519 | 0.7169±0.04399 | 0.6615±0.09802 | 0.6001±0.06699 | 0.6255±0.06803 |
| LVAW-s (mm) | 1.002±0.07925 | 1.069±0.05807 | 1.164±0.05955 | 0.9342±0.1289 | 0.8575±0.09514 | 0.9755±0.1019 |
| LVID-d (mm) | 3.344±0.07126 | 3.396±0.07681 | 3.625±0.1375 | 3.702±0.1438 | 3.554±0.2003 | 3.325±0.1490 |
| LVID-s (mm) | 2.007±0.1078 | 2.136±0.1388 | 2.355±0.1753 | 2.950±0.1470* | 2.686±0.2395 | 2.333±0.1494 |
| LVPW-d (mm) | 0.7798±0.09599 | 0.8643±0.1227 | 0.8348±0.1298 | 0.7646±0.1014 | 0.6173±0.04489 | 0.6611±0.04004 |
| LVPW-s (mm) | 1.381±0.1557 | 1.320±0.1572 | 1.243±0.1108 | 0.9523±0.08394 | 0.8676±0.08626* | 0.9625±0.08311 |
| LVEF (%) | 73.48±2.341 | 70.76±3.430 | 71.19±2.930 | 47.59±3.318** | 46.82±3.351** | 62.28±1.931^#&&^ |
| LVFS (%) | 41.54±2.162 | 39.63±2.901 | 39.83±2.460 | 23.50±1.897** | 22.95±1.834** | 32.76±1.330^#&^ |

*P<0.05 vs. Control; **P<0.01 vs. Control; ^#^P<0.05 vs. DOX; ^##^P<0.01 vs. DOX; ^&^P<0.05 vs. DOX+rAAV9-GFP; ^&&^P<0.01 vs. DOX+rAAV9-GFP.

**Supplementary Table 6. Data of hemodynamic analyses of mice injected with adeno-associated virus.**

|  | **Control** | **rAAV9-GFP** | **rAAV9-PPARα** | **DOX** | **DOX+rAAV9-GFP** | **DOX+rAAV9-PPARα** |
| --- | --- | --- | --- | --- | --- | --- |
| Max dp/dt (mmHg/s) | 7352±1129 | 5620±98.10 | 5935±767.8 | 2655±67.47** | 2443±365.7** | 6091±763.6^#&^ |
| Min dp/dt (mmHg/s) | -5405±748.1 | -4567±471.3 | -3786±285.5 | -2146±455.6** | -1989±266.9** | -4533±677.6^&^ |

*P<0.05 vs. Control; **P<0.01 vs. Control; ^#^P<0.05 vs. DOX; ^##^P<0.01 vs. DOX; ^&^P<0.05 vs. DOX+rAAV9-GFP; ^&&^P<0.01 vs. DOX+rAAV9-GFP.
